# Fine-scale structures as spots of increased fish concentration in the open ocean

**DOI:** 10.1038/s41598-021-94368-1

**Published:** 2021-08-04

**Authors:** Alberto Baudena, Enrico Ser-Giacomi, Donatella D’Onofrio, Xavier Capet, Cedric Cotté, Yves Cherel, Francesco D’Ovidio

**Affiliations:** 1 Sorbonne Université, CNRS, IRD, MNHN, Laboratoire d’Océanographie et du Climat: Expérimentations et Approches Numériques (LOCEAN-IPSL), Paris, France; 2 Sorbonne Université,CNRS, Laboratoire d’Océanographie de Villefranche, UMR 7093 LOV, Villefranche-sur-Mer, France; 3 Institute of Atmospheric Sciences and Climate, National Research Council (CNR-ISAC), Torino, Italy; 4 Copernicus Institute of Sustainable Development, Utrecht University, Utrecht, The Netherlands; 5 Centre d’Etudes Biologiques de Chizé (CEBC), UMR 7372 du CNRS-La Rochelle Université, 79360 Villiers-en-Bois, France; 6 Department of Earth, Atmospheric and Planetary Sciences, Massachusetts Institute of Technology, 02139 Cambridge, MA USA

**Keywords:** Marine biology, Physical oceanography, Fisheries

## Abstract

Oceanic frontal zones have been shown to deeply influence the distribution of primary producers and, at the other extreme of the trophic web, top predators. However, the relationship between these structures and intermediate trophic levels is much more obscure. In this paper we address this knowledge gap by comparing acoustic measurements of mesopelagic fish concentrations to satellite-derived fine-scale Lagrangian Coherent Structures in the Indian sector of the Southern Ocean. First, we demonstrate that higher fish concentrations occur more frequently in correspondence with strong Lagrangian Coherent Structures. Secondly, we illustrate that, while increased fish densities are more likely to be observed over these structures, the presence of a fine-scale feature does not imply a concomitant fish accumulation, as other factors affect fish distribution. Thirdly, we show that, when only chlorophyll-rich waters are considered, front intensity modulates significantly more the local fish concentration. Finally, we discuss a model representing fish movement along Lagrangian features, specifically built for mid-trophic levels. Its results, obtained with realistic parameters, are qualitatively consistent with the observations and the spatio-temporal scales analysed. Overall, these findings may help to integrate intermediate trophic levels in trophic models, which can ultimately support management and conservation policies.

## Introduction

Marine biomass distribution is highly patchy and variable in time across the entire trophic web^1,2^. Discerning the factors underpinning ocean patchiness is fundamental to understand how they influence biogeochemical reactions and ecosystem stability^3,4^. These issues are pivotal for conservation purposes^5^ and for assessing the impact of climate change on the marine environment^6^.

One of the origins of the heterogeneity of biotic fields is the dynamic nature of ocean environments, which transports water masses and affects their properties on a large range of temporal scales, including those of ecological relevance. In this regard, the mesoscale and submesoscale processes^7,8^, now commonly referred to together as “fine-scales” (from a few to hundreds of kilometers) influence importantly the ecological landscape. One fruitful approach for capturing the structuring effect of fine-scale dynamics is the extraction of so-called Lagrangian Coherent Structures, (LCS^9,10^). LCSs provide several types of information regarding flow properties, such as the location of barriers to transport^11^, or retentive and coherent regions^12^. In particular, LCSs permit to identify frontal features (hereafter, Lagrangian fronts), usually associated with the presence of strong environmental gradients (see^13^ for a strict definition). One of the most common Lagrangian diagnostics used to determine LCSs is the Finite-size Lyapunov Exponent (FSLE^14^). This quantity measures the exponential rate of water parcel deformation and has maximal values (ridges) over frontal regions.

By shaping and elongating water patches, Lagrangian Coherent Structures have been demonstrated to set the frontiers of phytoplanktonic patches in terms of chlorophyll concentration^10^, and even functional type^15^. This in turn enhances contacts between different communities, regulating plankton diversity^16,17^.

More recently, advances in biologging programs provided evidence on the impact of fine-scale structures on top predators behavior. The concentration of predators foraging efforts has been observed in association with Lagrangian fronts^18,19^. Furthermore, fronts detected by Lagrangian Coherent Structures have been observed to influence predators movements^20^. This could enhance energy transfer and gain^21^.

However, while the influence of Lagrangian fronts has been observed on both extremes of the trophic web, much less is known about mid-trophic levels. With this term we will refer to micronektonic organisms such as mesopelagic fish, which are able to actively move along the horizontal. Prants et al. (see in particular^22^) demonstrated a correlation between Pacific saury catches and Lyapunov exponents, and^23^ found that several fishery vessels track LCSs when targeting fishery spots. However, these results leave some concerns about possible biases because commercial fisheries provides only isolated observations. In addition, fishing vessels may intentionally target frontal systems through the satellite images they are known to use. Unbiased fish measurements have been instead recently used by^24^ to analyse the relationship between a frontal system and acoustic measurements in a coastal upwelling system. This allowed the authors to highlight the different role played by in-shore and off-shore waters. In terms of the mechanisms which can explain how fine-scale structures influence mid-trophic biomass distribution, even less is known. Classical explanations are based primarily on bottom-up mechanisms along fronts with intense upwelling^8,25,26^. However, these hypotheses do not take into account the necessity of a maturation time, which in the case of fish is consistently longer than both the growth response of lower trophic levels (such as phyto-and zooplankton) and the lifetime of the front^27,28^. Nor is the fish behavior considered, despite the fact that fish possess efficient sensorial and horizontal swimming capacities (^29^ and Supporting Information SI.2).

Note that fine-scale dynamics include mixed-layer frontal activity generated by kilometer-scale instability processes^30^. These instabilities are a priori unlikely to influence mesopelagic fish because by definition they are restricted to the mixed layer^8^. Therefore, we consider here only the deep-reaching fronts associated with mesoscale stirring captured by altimetry-derived Lagrangian methods.

This study was conducted in the subantarctic area of the Southern Indian Ocean. The functioning of this region is mainly regulated by the Kerguelen plateau, a major topographic barrier for the Antarctic Circumpolar Current (ACC). The plateau fertilizes in iron, a limiting nutrient, the so-called “high-nutrients-low-chlorophyll” waters advected by the ACC^31^. Depending on seasonal light conditions and stratification of the water column, this provokes a large annual phytoplanktonic bloom, which supports a rich trophic web. This is one of the reasons for which the Kerguelen archipelago and its surrounding waters are part of one of the ten largest marine protected areas in the world (http://www.mpatlas.org/).

In this region, myctophids, also known as lantern fish, are one of the most abundant groups of mesopelagic fish. They are also present in other oceans worldwide and are thought to constitute one the largest portions of world fish biomass^32^. They also represent important prey items for numerous predators^33^. Myctophids are reported to play a central role in the carbon export to deep sea depths, and are suspected to affect the climate^34^. Constituting a potentially massive harvestable resource, they are threatened to be exploited in the near future^35^. In addition, their description in trophic models should be improved, and a better understanding of the mechanisms regulating their biomass is pivotal for fisheries management and sound marine spatial planning^36^.

The objective of the present study is to analyze the relationship between fine-scale structures in the open ocean and mid-trophic levels with unbiased, direct observations of fish concentrations. More specifically, the aim is to explore the degree to which fish distribution is shaped by fine-scale features. In order to achieve this, we firstly analyze the difference in fish concentrations observed in presence or absence of fronts. Secondly, we investigate the relationship between the intensity of the frontal dynamics and the fish distribution. Third, we examine this relationship on chlorophyll-rich waters only. Finally, we propose a mechanism by which fine-scale processes could potentially organize mid-trophic level biomass. This model considers explicitly active fish movement, and is calibrated with realistic, non-fitted parameters, derived from observations. The objective of the model is to assess (i) whether the modeled fish concentration varies significantly in presence of frontal features and (ii) if the spatio-temporal scales of the modeled process are comparable with those observed in the present study.

## Materials and methods

### Acoustic measurements

A set of acoustic echo sounder data was used to analyze fish density. This was collected within the Mycto-3D-MAP program using split-beam echo sounders at 38 and 120 kHz. The Mycto-3D-MAP program included multiple large-scale oceanographic surveys during 2 years and a dedicated cruise in the Kerguelen area. The dataset was collected during 4 large-scale surveys in 2013 and 2014, both in summer (including both northward and southward transects) and in winter, corresponding to 6 acoustic transects of 2860 linear kilometers (see Table 1 for more details). Note that all legs except summer 2014 (MYCTO-3D-Map cruise) were logistic operations, during which environmental in situ data (such as temperature or salinity profiles) could not be collected. The data were then treated with a bi-frequency algorithm, applied to the 38 and 120 kHz frequencies (details of data collection and processing are provided in^37^). This provides a quantitative estimation of the concentration of gas-bearing organisms, mostly attributed to fish containing a gas-filled swimbladder in the water column^38^. Most mesopelagic fish present swimbladders and several works pointed out that myctophids are the dominant mesopelagic fish in the region^39^. Therefore, we considered the acoustic signal as mainly representative of myctophids concentration. Data were organized in acoustic units, averaging acoustic data over 1.1 km along the ship trajectory on average. Myctophid school length is in the order of tens of meters^40^. For this reason, acoustic units were considered as not autocorrelated. Every acoustic unit is composed of 30 layers, ranging from 10 to 300 meters (30 layers in total).

The dataset was used to infer the Acoustic Fish Concentration (AFC) in the water column. We considered as AFC of the point ($x_i$, $y_i$) of the ship trajectory the average of the bifrequency acoustic backscattering of 29 out of 30 layers (the first layer, 0-10 m, was excluded due to surface noise) AFC quantity is dimensionless.

As the previous measurements were performed through acoustic measurements, a non-invasive technique, fishes were not handled for this study.

**Table 1:** Details of the acoustic transects analyzed.

**Acoustic campaigns details**				
Cruise	Season	St. Date	End Date	Distance (km)
LOGIPEV193_RUNKER	Summer	09/02/2013	17/02/2013	2752
LOGIPEV193_KERMAU	Summer	04/03/2013	10/03/2013	3781
OP2013-2_RUNKER	Winter	30/08/2013	10/09/2013	3310
LOGIPEV197_RUNKER	Summer	06/01/2014	13/01/2014	2800
LOGIPEV197_KERMAU	Summer	06/02/2014	18/02/2014	2045
OP2014-2_RUNKER	Winter	24/08/2014	04/09/2014	3677

### Regional data selection

The geographic area of interest of the present study is the Southern Ocean. To select the ship transects belonging to this region, we used the ecopartition of^41^. Only points falling in the *Antarctic Southern Ocean* region were considered. We highlight that this choice is consistent with the ecopartition of^42^ (group 5), which is specifically designed for myctophids, the reference fish family (Myctophidae) of this study. Furthermore, this choice allowed us to exclude large scale fronts (i.e., fronts that are visible on monthly or yearly averaged maps) which have been the subject of past research works^43,44^. In this way our analysis is focused specifically on fine-scale fronts.

### Day-night data separation

Several species of myctophids present a diel vertical migration. They live at great depths during the day (between 500 and 1000 m), ascending at dusk in the upper euphotic layer, where they spend the night. Since the maximal depth reached by the echo sounder we used is 300 m, in the analysis reported in Figs. 2 and 3 we excluded data collected during the day. However, their analysis is reported in SI.1. A restriction of our acoustic analyses to the ocean upper layer is also consistent with the fact that the fine-scale features we computed are derived in this work by satellite altimetry, thus representative of the upper part of the water column ($\sim 50$ m). Finally, we note that the choice of considering the echo sounder data in the first 300 m of the water columns is coherent with the fact that LCS may extend almost vertically in depth even at 600 m depth^45,46^ and with the fact that altimetry-derived velocity fields are consistent with subsurface currents in our region of interest down to 500 m^20^.

### Satellite data

*Velocity current data and Finite-Size Lyapunov Exponent (FSLE) processing.* Velocity currents are obtained from Sea Surface Height (SSH), which is measured by satellite altimetry, through geostrophic approximation. Data, which were downloaded from E.U. Copernicus Marine Environment Monitoring Service (CMEMS, http://marine.copernicus.eu/), were obtained from DUACS (Data Unification and Altimeter Combination System) delayed-time multi-mission altimeter, and displaced over a regular grid with spatial resolution of $\frac{1}{4}\times \frac{1}{4}^\circ$ and a temporal resolution of 1 day.

Trajectories were computed with a Runge-Kutta scheme of the 4th order with an integration time of 6 hours. Finite-size Lyapunov Exponents (FSLE) were computed following the methods in^14^, with initial and final separation of $0.04^\circ$ and $0.4^\circ$ respectively. This Lagrangian diagnostic is commonly used to identify Lagrangian Coherent Structures. This method determines the location of barriers to transport, and it is usually associated with oceanic fronts^9^. Details on the Lagrangian techniques applied to altimetry data, including a discussion of its limitation, can be found in^10^.

#### Temperature field and gradient computation

 The Sea Surface Temperature (SST) field was produced from the OSTIA global foundation Sea Surface Temperature (product id: SST_GLO_ SST_L4_NRT_OBSERVATIONS_010_001) from both infrared and microwave radiometers, and downloaded from CMEMS website. The data are represented over a regular grid with spatial resolution of $0.05\times 0.05^\circ$ and daily-mean maps. The SST gradient was obtained from:

$$\begin{aligned} Grad SST=\sqrt{g_x^2+g_y^2} \end{aligned}$$

where $g_x$ and $g_y$ are the gradients along the west-east and the north-south direction, respectively. To compute $g_x$, the following expression was used:

$$\begin{aligned} g_x=\frac{1}{2 dx}\cdot (SST_{i+1}-SST_{i-1}) \end{aligned}$$

where the SST values of the adjacent grid cells (along the west-east direction: cells $i+1$ and $i-1$) were employed. *dx* identifies the kilometric distance between two grid points along the longitude (which varies with latitude). The definition is analog for $g_y$, considering this time the north-south direction and $dy\simeq 5$ km (0.05$^\circ$).

#### Chlorophyll field

 Chlorophyll estimations were obtained from the Global Ocean Color product (OCEANCOLOUR_ GLO_CHL_L4_REP_OBSERVATIONS_009_082-TDS) produced by ACRI-ST. This was downloaded from CMEMS website. Daily observations were used, in order to match the temporal resolution of the acoustic and satellite observations. The spatial resolution of the product is 1/24$^{\circ }$.

#### Estimation of satellite data along ship trajectory

 For each point ($x_i$, $y_i$) of the ship trajectory, we extracted a local value of FSLE, SST gradient, and chlorophyll concentration. These were obtained by considering the respective average value in a circular around of radius $\sigma$ of each point ($x_i$, $y_i$) . Different $\sigma$ were tested (ranging from 0.1$^\circ$ to 0.6$^\circ$), and the best results were obtained with $\sigma =0.2^\circ$, reference value reported in the present work. This value is consistent with the resolution of the altimetry data.

### Statistical processing

#### Front identification

 FSLE and SST gradient were used as diagnostics to detect frontal features, since they are typically associated with front intensity and Lagrangian Coherent Structures^10^. Note that the two diagnostics provide similar but not identical information. FSLEs are used to analyze the horizontal transport and to identify material lines along which a confluence of waters with different origins occur. These lines typically display an enhanced SST gradient because water masses of different origin have often contrasted SST signature. However, this is not a necessary condition. FSLE ridges may be invisible in SST maps if transport occurs in a region of homogeneous SST. Conversely, SST gradient unveils structures separating waters of different temperatures, whose contrast is often – but not always – associated with horizontal transport. Therefore, even if they usually detect the same structures, these two metrics are complementary. Frontal features were identified by considering a local FSLE (or SST gradient, respectively) value larger than a given threshold. The threshold values have been chosen heuristically but consistently with the ones found in previous works. For the FSLEs, we used 0.08 days$^{-1}$, a threshold value in the range of the ones chosen in^18^ and^47^. For the SST gradient, we considered representative of thermal front values greater than 0.009${^\circ }$C/km, which is about half the value chosen in^47^. However, in that work, the SST gradient was obtained from the advection of the SST field with satellite-derived currents for the previous 3 days, a procedure which is known to enhance tracer gradients.

#### Bootstrap method

 The threshold value splits the AFC into two groups: AFC co-located with FSLE or SST gradient values over the threshold are considered as measured in proximity of a front (i.e., statistically associated with a front), while AFC values below the threshold are considered as not associated with a frontal structure. The statistical independence of the two groups was tested using a Mann-Whitney or U test. Finally, bootstrap analysis is applied following the methodologies used in^47^. This allows estimating the probability that the difference in the mean AFC values, over and under the threshold, is significant, and not the result of statistical fluctuations. Other diagnostics tested are reported in SI.1.

#### Linear quantile regression

 Linear quantile regression method^48^ was employed as no significant correlation was found between the explanatory and the response variables. This can be due to the fact that multiple factors (such as prey or predator distributions) influence the fish distribution other than the frontal activity considered. The presence of these other factors can shadow the relationship of the explanatory variables (in this case, the FSLE and the SST gradient) with the mean value of the response variable (the AFC). A common method to address this problem is the use of the quantile regression^48,49^, that explores the influence of the explanatory variables on other parts of the response variable distribution. Previous studies, adopting this methodology, revealed the limiting role played by the explanatory variables in the processes considered^50^. The percentiles values used are 75th, 90th, 95th, and 99th. The analysis is performed using the statistical package QUANTREG in R (https://CRAN.R-project.org/package=quantreg, v.5.38^48,51^), using the default settings.

#### Chlorophyll-rich waters selection

 The AFC observations were considered in chlorophyll-rich waters if the corresponding chlorophyll concentration was higher than a given threshold. All the other AFC measurements were excluded, and a linear regression performed between the remaining AFC and FSLE (or SST gradient) values. The corresponding thresholds (one for FSLE and one for SST gradient case) were selected though a sensitivity test reported in SI.1. These resulted in 0.22 and 0.17 mg/m$^3$ for FSLE and for SST gradient, respectively. These values are consistent among them and, in addition, they are in coherence with previous estimates of chlorophyll concentration used to characterise productive waters in the Southern Ocean (0.26mg/m$^3$^52^).

### Gradient climbing model

An individual-based mechanistic model is built to describe how fish could move along frontal features. We assume that the direction of fish movement along a frontal gradient is influenced by the noise of the prey field (SI. 2). Specifically, we consider a Markovian process along the (one dimensional) cross-front direction. Time is discretized in timesteps of length $\varDelta \tau$. We presuppose that, at each timestep, the fish chooses between swimming in one of the two opposite cross-front directions (“left” and “right”). This decision depends on the comparison between the quantity of a tracer (a cue) present at its actual position and the one perceived at a distance $p_R$ from it, where $p_R$ is the perceptual range of the fish. We consider the fish immersed in a tracer whose concentration is described by the function *T*(*x*).

An expression for the average velocity of the fish, $U_F(x)$, can now be derived. We assume that the fish is able to observe simultaneously the tracer to its right and its left. Fish sensorial capacities are discussed in SI.2. The tracer observed is affected by noise. Noise distribution is considered uniform, with $-\xi _{MAX}<\xi <\xi _{MAX}$, $\xi _{MAX}>0$.

 The effective values perceived by the fish, at its left and its right, will be, respectively:

$$\begin{aligned}&{\tilde{T}}(x_0-\varDelta x)=T(x_0-\varDelta x)+\xi _1 \\&{\tilde{T}}(x_0+\varDelta x)=T(x_0+\varDelta x)+\xi _2 \;. \end{aligned}$$.

We assume that, if ${\tilde{T}}(x_0+\varDelta x)>{\tilde{T}}(x_0-\varDelta x)$, the fish will move to the right, and, vice versa, to the left. We hypothesize that the observational range is small enough to consider the tracer variation as linear, as illustrated in Fig. S7 (SI. 3). In this way:

$$\begin{aligned}&{\tilde{T}}(x_0+\varDelta x)=T(x_0)+ p_R\,\frac{\partial T}{\partial x}+\xi _1 \\&{\tilde{T}}(x_0-\varDelta x)=T(x_0)- p_R\,\frac{\partial T}{\partial x}+\xi _2 \;. \end{aligned}$$

In case of absence of noise, or with $\xi _{MAX}<p_R\,\frac{\partial T}{\partial x}$, the fish will always move in the correct direction, in that it will climb the gradient. Assuming *V* as the cruising swimming velocity of the fish, this means $U_F(x)=V$.

Let’s now assume $\xi _{MAX}>p_R\,\frac{\partial T}{\partial x}$. If $T(x_0+\varDelta x)>T(x_0-\varDelta x)$ (as in Fig. S7), and the fish will swim leftward if

$$\begin{aligned} \xi _1-\xi _2>2p_R\,\frac{\partial T}{\partial x}\; . \end{aligned}$$

Since $\xi _1$ and $\xi _2$ range both between $-\xi _{MAX}$ and $\xi _{MAX}$, we can obtain the probability of leftward moving *P*(*L*). This will be the probability that the difference between $\xi _1$ and $\xi _2$ is greater than $2p_R\,\frac{\partial T}{\partial x}$

$$\begin{aligned} P(L)&=\frac{1}{8\xi _{MAX}^2} \bigg (2 \xi _{MAX} - 2 p_R\,\frac{\partial T}{\partial x}\bigg )^2\\&=\frac{1}{2} \bigg (1-\frac{p_R}{\xi _{MAX}}\,\frac{\partial T}{\partial x}\bigg )^2 \end{aligned}$$.

The probability of moving right will be

$$\begin{aligned} P(R)&=1-P(L) \end{aligned}$$

and their difference gives the frequency of rightward moving

$$\begin{aligned} P(R)-P(L)&=1-2P(L)=1-\bigg (1-\frac{p_R}{\xi _{MAX}}\,\frac{\partial T}{\partial x}\bigg )^2\\&=\frac{p_R}{\xi _{MAX}}\frac{\partial T}{\partial x}\bigg (2-\frac{p_R}{\xi _{MAX}}\bigg |\frac{\partial T}{\partial x}\bigg |\bigg )\; , \end{aligned}$$

where the absolute value of $\frac{\partial T}{\partial x}$ has been added to preserve the correct climbing direction in case of negative gradient. The above expression leads to:

1 $$\begin{aligned} U_F(x)=\frac{V p_R}{\xi _{MAX}}\frac{\partial T}{\partial x}\bigg (2-\frac{p_R}{\xi _{MAX}}\bigg |\frac{\partial T}{\partial x}\bigg |\bigg )\;. \end{aligned}$$

We then assume that, over a certain value of tracer gradient $\frac{\partial T}{\partial x}_{MAX}$, the fish are able to climb the gradient without being affected by the noise. This assumption, from a biological perspective, means that the fish does not live in a completely noisy environment, but that under favorable circumstances it is able to correctly identify the swimming direction that leads to higher prey availability. This means that

2 $$\begin{aligned} p_R*\frac{\partial T}{\partial x}_{MAX}=\xi _{MAX}\,. \end{aligned}$$

Substituting then (2) into (1) gives:

3 $$\begin{aligned} U_F(x)=V \frac{\frac{\partial T}{\partial x}}{\frac{\partial T}{\partial x}_{MAX}}\bigg (2-\frac{\big |\frac{\partial T}{\partial x}\big |}{\frac{\partial T}{\partial x}_{MAX}}\bigg )\;. \end{aligned}$$

Finally, we can include an eventual effect of transport by the ocean currents, considering that the tracer is advected passively by them, simply adding the current speed $U_C$ to Expr. (3).

The representations provided are individual based, with each individual representing a single fish. However, we note that all the considerations done are also valid if we consider an individual representing a fish school. $U_F$ will then simply represent the average velocity of the fish schools. This aspect should be stressed since many fish species live and feed in groups, especially myctophids (see SI.2 for further details).

#### Continuity equation in one dimension

 The solution of this model will now be discussed. The continuity equation is first considered in one dimension. The starting scenario is simply an initially homogeneous distribution of fish, that are moving in a one dimensional space with a velocity given by $U_{F}(x)$.

We assume that in the time scales considered (few days to some weeks), the fish biomass is conserved, so for instance fishing mortality or growing rates are neglected. In that case, we can express the evolution of the concentration of the fish $\rho$ by the continuity equation

4 $$\begin{aligned} \frac{\partial \rho }{\partial t}+\nabla \cdot \mathbf{j }\,=\,0 \end{aligned}$$

in which $\mathbf{j }=\rho \;U_{F}(x)$, so that Eq. (4) becomes

5 $$\begin{aligned} \frac{\partial \rho }{\partial t}+\nabla \cdot \big (\rho \;U_{F}(x)\big )\,=\,0\;. \end{aligned}$$

In one dimension, the divergence is simply the partial derivate along the *x-*axis. Eq. (5) becomes

6 $$\begin{aligned} \frac{\partial \rho }{\partial t}=-\frac{\partial }{\partial x} \bigg (\rho \;U_{F}\bigg ) \end{aligned}$$

Now, we decompose the fish concentration $\rho$ in two parts, a constant one and a variable one $\rho \,=\,\rho _0+{\tilde{\rho }}$. Eq. (6) will then become

7 $$\begin{aligned} \frac{\partial \rho }{\partial t}=-U_F\frac{\partial {\tilde{\rho }}}{\partial x}-\rho \frac{\partial U_F}{\partial x}\;. \end{aligned}$$

Using Expr. (3), Eq. (7) is numerically simulated with the Lax method. In Expr. (3) we impose that $U_F(x)=V$ when $U_F(x)>V$.

 This biological assumption means that fish maximal velocity is limited by a physiological constraint rather than by gradient steepness. Details of the physical and biological parameters are provided in SI.6.

## Results

### Relationships between acoustic fish concentration and satellite-derived diagnostics

**Figure 1:**
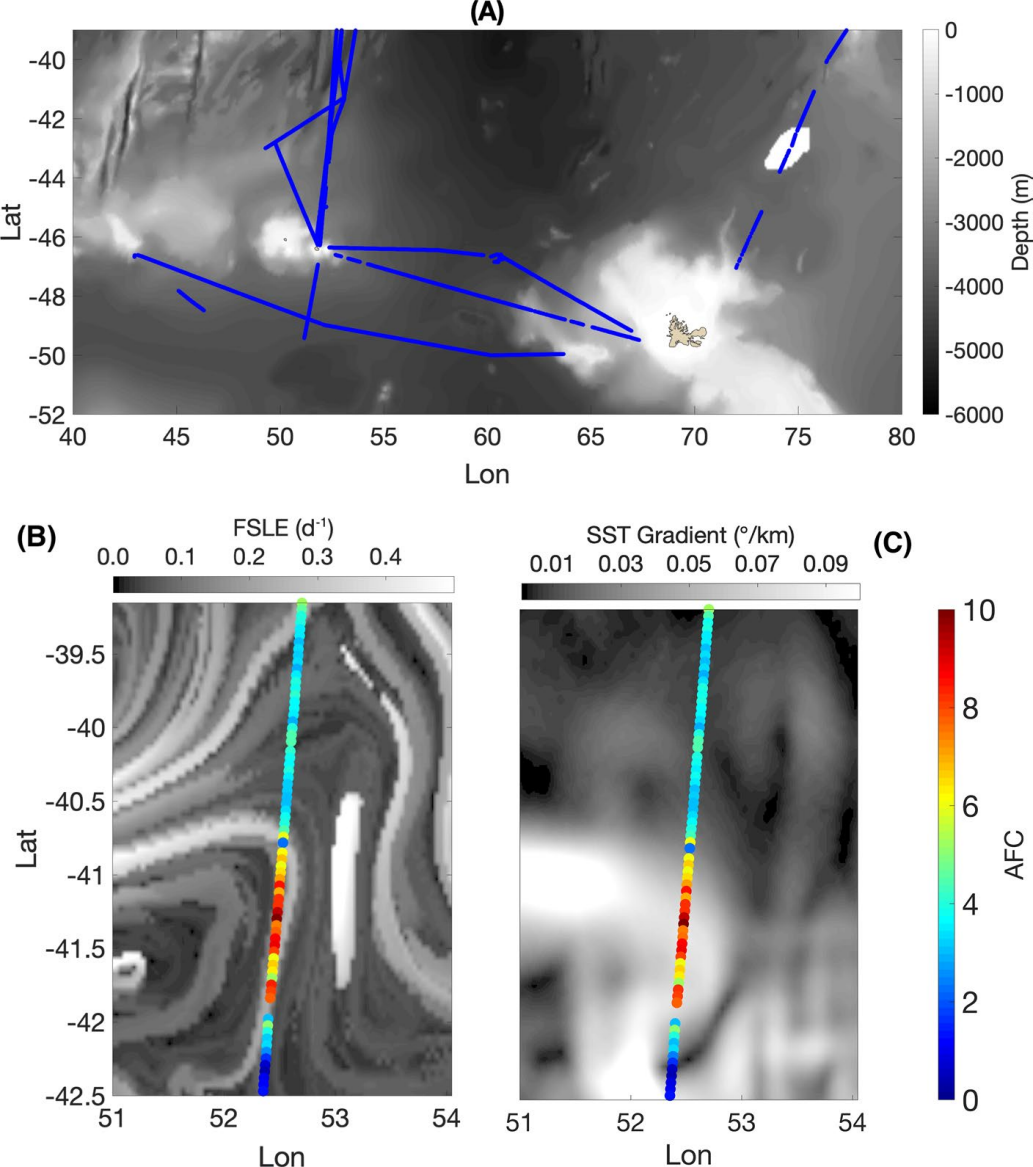
**(A)** Overview of the study region. Blue dots show the position of the survey transects conducted during the Mycto-3D-MAP program. They are superimposed on the local bathymetry (gray scale at right). **(B)** Illustrative example of a transect of the ship trajectory on August, 29th, 2014, superimposed on a simultaneous field of Finite-size Lyapunov Exponents (i.e., frontal structures; upper gray scale). The color of each dot is proportional to the local Acoustic Fish Concentration (adimensional, scale at right). **(C)** Same as for panel (**B**), with the difference that the transect is superimposed on a simultaneous field of Sea Surface Temperature gradient (upper gray scale). Note that panels (**B**) and (**C**) show a region at the border of the domain analysed.

A good qualitative agreement is found when comparing the AFC along some portions of the ship transect with the local FSLE and SST gradient fields (Fig. 1). AFCs seem to increase in correspondence with the frontal features identified by high values of the Lyapunov exponents and temperature gradient. A visual exploration of the relation between AFC and ridges of in the FSLE field is difficult to interpret, due to the intermittent nature of the AFC and to the fact that the ship transects intersect FSLE ridges with various angles.

#### Bootstrap analysis

**Figure 2:**
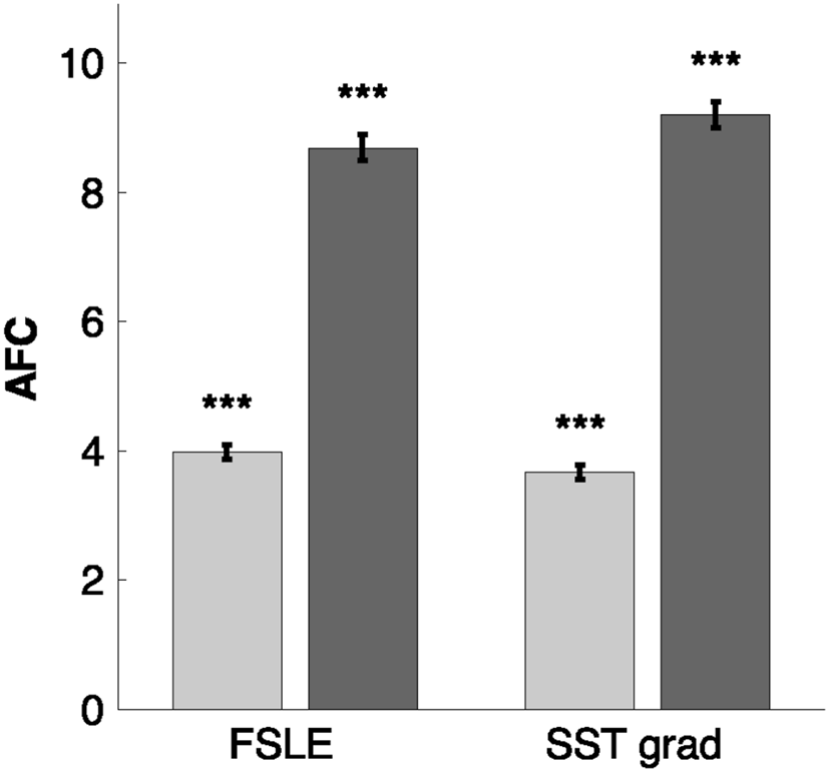
Bootstrap method results. AFC values were separated according to the respective FSLE and SST gradient values, using a threshold as a limit to define a front (0.08 days$^{-1}$ and 0.009${^\circ }$C/km respectively;^18,47^) Light gray columns represent the mean AFC under the respective threshold, considered outside a front. Dark gray columns represent the mean AFC over the threshold, thus in proximity of a front. Left columns refer to FSLE analysis, right columns to SST gradient. Error bars indicate the standard deviation, while black stars indicate the significance of the bootstrap test (***: *p*<0.001).

In order to quantify whether the AFC values present significant differences in proximity of fine-scale features, a bootstrap analysis was conducted. Therefore, AFCs were separated in two groups: those falling over a front, identified by FSLE (or SST gradient) values over a threshold, and those falling outside it (further details are provided in Materials and Methods). Bootstrap analysis indicates that significantly higher AFC values are detected in presence of fronts (*p*-value < 0.001) for both criteria of front detection (FSLE or SST gradient, Fig. 2). This result demonstrates that high fish concentrations occur preferably over fronts detected with the diagnostics employed.

#### Quantile regression analysis

**Figure 3:**
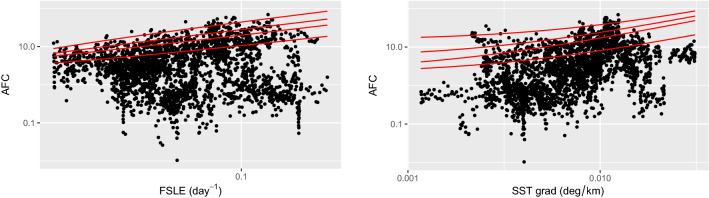
Scatter plot of AFC against FSLE (left panel) and SST gradient (right panel) for all the points sampled (thus not filtered based on chlorophyll). The lines, from the bottom to the top, indicate the linear quantile regressions at 75th, 90th, 95th and 99th percentiles. The analysis is used to investigate the relationship between the front intensity and just the higher values of fish concentration. Both axes are in logarithmic scale (values equal to zero are therefore not depicted). Values of the quantile regression coefficients are reported in Table S1.

 Subsequently, we analyzed the relationship between the intensity of the fronts (provided by the FSLE and SST gradient) and the AFC. When interpolating the data with a linear fit, no significant correlation was found between AFC values and FSLE or SST gradient. Therefore, linear quantile regression method was applied using the 75th, 90th, 95th, and 99th percentiles (Fig. 3; refer to Materials and Methods for further details). All the quantile regression slopes are significantly different from zero (Table S1 and sensitivity test in SI.1), meaning that FSLE and SST gradient intensities play a role in limiting fish concentration. This means that large fish concentrations are associated with the nearby presence of a strong FSLE or SST gradient value. Conversely, not all of the strong fronts are always associated with large fish densities.

#### Chlorophyll-rich waters analysis

**Figure 4:**
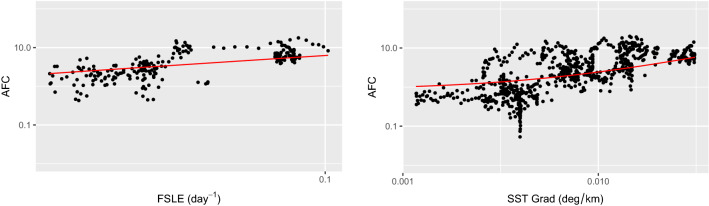
Left panel: scatter plot of AFC against FSLE (left panel) for the sub-set of points whose local chlorophyll concentration is higher than 0.22 mg/m$^3$. Right panel: scatter plot of AFC against SST gradient (right panel) for the sub-set of points whose local chlorophyll concentration is higher than 0.17 mg/m$^3$. For both panels, the red line is obtained from the respective linear interpolation of the selected points (note that axes are reported in logarithmic scale). Left panel: R = 0.66, R$^2$ = 0.43, *p* < 0.001. Right panel: R = 0.54, R$^2$ = 0.29, *p* < 0.001. Sensitivity of the results with respect to the chlorophyll threshold chosen is reported in SI.1.

 The previous results evidence that, together with front intensity, other unaccounted factors play a role in shaping fish distribution. In the following, we investigate the importance of one of these elements, notably the presence of prey. We use the local chlorophyll concentration as a proxy to determine whether a front is found in a region potentially rich in prey for fish or not. This choice is driven by the fact that areas with enhanced primary production are also known to host higher trophic organisms^27,28^ such as zooplankton (a prey for myctophids). Therefore, we consider only the points of the ship trajectory whose local value of chlorophyll is larger than a threshold. The threshold value is identified through a sensitivity test in SI.1, and it is found consistent for both FSLE and SST gradient. When this selection is applied, a significant linear relationship emerges between the AFC and the intensity of the frontal dynamics (Fig. 4; FSLE: R = 0.66,* p* < 0.001; SST gradient: R = 0.54,* p* < 0.001).

### A fine-scale mechanism of fish aggregation

Why do fish aggregate along frontal features? To try to address this question, we propose a simple mathematical model (see “Gradient climbing model” section for details). The model assumes that fish have a gradient climbing capacity, which is one of the most widespread movement mechanisms used in other biological contexts (e.g., chemotaxis^53^). This gradient climbing capacity is specifically tuned for mid-trophic levels and myctophids and is based on a cue-pursuing dynamic. Fish try to climb a gradient of tracer. We considered this tracer a proxy of zooplankton concentration, main prey of several fish species, including myctophids^54^. At the scales considered in this study (10s of kilometers) zooplankton swimming capacities are restricted to the vertical axis^55^. They can thus be considered as passive tracers along the horizontal. Along this spatial dimension, zooplankton aggregation and growth is usually driven by a relatively fast response to nutrients presence, in the order of days to weeks^27,28,56^. In particular, this is valid also for zooplankton species present in our study region^57,58^. Conversely, fish have growth rates consistently slower: in particular, pelagic fishes and myctophids are considered as “slow-growing fish”, with lifetimes spanning a few years^59^. Therefore, aggregation can not be explained by a quick increase of their biomass. Alongside this, fish have extremely developed sensorial capacities and, in contrast to zooplankton, they can actively swim, with both capacities involved in many functional activities, including feeding^29^. These arguments support our approach of modelling zooplankton as a passive tracer and fish as active swimmers (we invite the reader to refer to SI.2 for further details).

To account for ocean patchiness and small scale turbulence, a noise term perturbed the ability of the fish to properly identify the elevated zooplankton regions. However, we assumed that fish are able to orientate without problems over a given threshold of the zooplankton gradient. This threshold was estimated from the zooplankton concentrations (SI.5).

**Figure 5:**
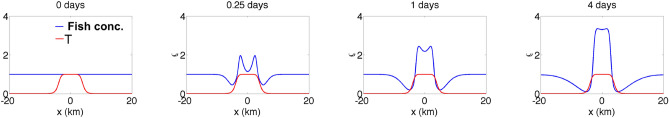
Time evolution of the fish concentration (blue line, adimensional) according to the continuity equation. The tracer (red line, adimensional) describes a plateau of 8 km in width. At its limits, its values range from 1 to 0 in about 5 km. Each panel represents a different snapshot at 0, 6 hours, 1 day, and 4 days.

**Figure 6:**
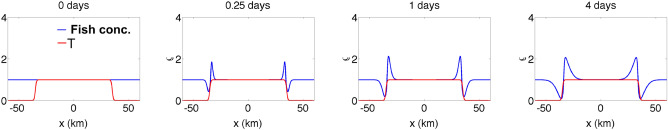
Time evolution of the fish concentration as reported in Fig. 5. This time, the plateau width has been set to 70 km.

The distribution of the tracer used as a proxy of zooplankton concentration follows a sigmoid function (red line in Figs. 5 and 6), which models a generic local gradient. The transition zones at the edges of the tracer plateau are $\sim$5 km wide, as typically found for fine-scale features such as eddies or upwelling structures. Conversely, the plateau presents a local maximum at its center. We consider the modeled gradient as representative of a frontal feature.

Four different snapshots (at 0, 6 hours, 1 day and 4 days) of the fish modelled concentration are illustrated in Figs. 5 and 6. By construction, fish displacements are most conspicuous in regions of elevated tracer gradients, i.e., at the plateau edges. Indeed, after only 6 hours, two peaks of doubled concentration are present in correspondence with the margins of the tracer plateau. In the following days, the two peaks decrease their growth rate, while the concentration between them increases until they merge together. The fish concentration is thus homogeneous over the plateau, presenting values between 2.5 and 3.5 times higher than the initial concentration. A larger plateau (Fig. 6) produces two peaks of fish concentration in correspondence with its edges, while the merging between the peaks occurs over longer timescales (not reported). Changing the type of fish behavior leads to similar results (see SI.3).

At the same time during which this mechanism occurs, the tracer can evolve. As a sensitivity test, we numerically analyzed a scenario in which the tracer, subjected to a typical frontal dynamics, is stretched in a filament and eroded by diffusion (SI.4). Using realistic bio-physical parameters representative of the study area, we found that these tracer dynamics do not compromise the aggregation mechanism presented above, but may even facilitate the aggregation of fish along frontal zones. Indeed, the model developed predicts several quantitative information, such as an estimate of the fish aggregation over time. The latter highlighted a final concentration, on average, an order of magnitude stronger than the initial one. In addition, it was possible to estimate a “fish concentration life time”. This quantity indicates the amount of time during which a group of fish is able to follow a patch of interest before it vanishes due to the frontal dynamics. The school life times we obtained ranged between 7 to 25 days, with an average value of around 2 weeks. Remarkably, this amount of time matches with that of fine-scale processes (one day to several weeks).

The observations from the acoustic echo sounder have a coverage in space and time that is too limited to be used to test the validity of the model outputs. However, we highlight that the model predictions, obtained from realistic parameters without optimisation or fitting, are coherent with the spatio temporal scale analysed. Finally, we note that the modeled aggregation can occur only within prescribed conditions, notably the presence of prey. This is consistent with the results of both bootstrap, quantile, and chlorophyll-rich waters regression analyses, which pointed to the fact that fronts are not the only factor structuring fish abundance.

## Discussion

In the present work, we compare in situ measurements of Acoustic Fish Concentration with satellite-derived frontal structures, which were associated with the presence of Lagrangian fronts (detected through FSLE ridges) and strong Sea Surface Temperature gradients. When comparing the AFC with the presence (or absence) of a front, our findings illustrate that high AFCs are mostly associated with important frontal structures (Fig. 2, *p*< 0.001 both for FSLE and SST gradient). Subsequently, we analyze the relationship between AFC and front intensity. In this regard, we applied a quantile regression to investigate the relationship of FSLE and SST gradient with the upper part of the AFC distribution. This approach is consistent with the assumption that AFC is affected by several other processes (such as predators or prey presence), other than the presence of fronts. All the percentiles used (75th, 90th, 95th, and 99th) resulted in a quantile slope significantly different from zero (Fig. 3 and Table S1). This allowed us to unveil the important role played by front intensity on AFC, which could not have been detected through a standard linear correlation. In this regard, the presence of fronts can be seen as a limiting condition for high fish concentrations. This means that strong fish aggregation is preconditioned by the intensity of a frontal feature. Conversely, not all of the strong fronts detected indicate high AFCs, since other processes are involved in shaping fish abundance. Therefore, we examined the effect of one of these factors, notably the presence of prey. This was identified by analysing chlorophyll fields, which were considered as proxy of potential fish preys (such as zooplankton). Indeed, fronts sustaining a strong primary production can support also large zooplankton densities^27,28^. When considering only the fronts which are co-located with the presence of potential fish prey (i.e., large chlorophyll concentrations), our results show that such fronts modulate significantly the concomitant fish distribution (*p*<0.001 for both FSLE and SST gradient, R=0.66 and R=0.54 respectively, Fig. 4).

The AFC response to frontal features cannot be explained with traditional mechanisms usually prescribed to lower trophic levels, such as rapid growth associated to the presence of nutrients, because fish have slower growth rates. To address this question, we proposed a gradient-climbing model specifically calibrated for the study of mid-trophic levels. Importantly, this model considers explicitly active fish movement which, to our knowledge, has not been included directly in the study of fish aggregation to date (e.g.^26,60^). Once parameterized with values typical for the study region, the model can produce fish behavior characterized by a quick response to gradient structures. Simulated peaks of doubled fish concentration appear after just 6 hours in response to a front, and grow afterwards with lifetimes of several days. Interestingly, fish do not converge at the center of the patch of interest, where the maximal concentration of the patch is found. When the width of the plateau is in the order of hundred kilometers (Fig. 6), two peaks of fish concentration are produced at its edges, where the tracer gradient is maximum. Instead, when the width of the plateau is in the order of ten kilometers (Fig. 5), after a few days fish tend to be homogeneously distributed over it. On-going studies on myctophids’ response to food concentration presuppose that myctophids, over a certain prey density, ingest always the same quantity of food (Holling type III functional response; A. Hulley, personal communication). Therefore, above this threshold, they are expected to be homogeneously distributed. This is in qualitative agreement with the fish distribution predicted by the model.

The proposed mechanism of aggregation needs two obvious initial conditions: the presence of fish, and the presence of a zooplankton patch. It is presumable that fine-scale fronts that lack one or both these conditions cannot act as aggregating spots. Furthermore, while we assumed that the aggregation occurs after a certain amount of time, the environment studied is dynamic. Thus, it is likely that the aggregation mechanism was observed during different stages. Therefore, our model suggests that not all of the strong fronts should present high fish concentrations, but that, conversely strong fish aggregation is preconditioned by the intensity of a frontal feature. This is in accordance with the results obtained from the quantile regression and the chlorophyll-rich water analyses.

Tracer patchiness is known to be associated with frontal features^10,25,61^. However, patchiness evolves, and under the double effect of stretching and diffusion, local gradients can be eroded. Thus, we tested the robustness of our model to this feature in SI.4. As in the former case, none of the parameters employed has been optimized nor fitted, but they all represent rigorous estimations of Southern Ocean physical and biological conditions, such as stretching and mixing rates or fish cruising speed. Results allowed us to estimate a typical lifetime for a fine-scale patch of around two weeks, much longer than the peak doubling time ($\sim$ 6 hours). This robustness of the lifetime of the fine-scale patch is consistent with the hypothesis of a fixed tracer assumed in the gradient climbing scenario. Furthermore, we demonstrated that the stretching and diffusion dynamics can potentially enhance fish aggregations. In addition, the model provided estimations of other aggregation dynamics, such as the dimensions of the aggregation patterns or its intensity, with timescales comparable with those of fine-scale processes.

The modeled patch of interest is considered as a proxy of zooplankton concentration. However, being parameterized as a passive tracer, it can be considered, more simply, as a generic water property detected by fish. Indeed, physical characteristics, such as temperature, have been proven to be used by predators to find favorable conditions where concentrated food occurs^62^.

Dedicated missions are required to quantitatively validate the model results. This is currently not possible due to the sparsity, in time and space, of our acoustic dataset. First, continuous measurement are necessary to follow the potential aggregating mechanism along its different phases. Secondly, the displacement of the observed water mass, during the sampling period, must be taken into account.

Within the ACC, the information available on mid-trophic levels reported that the large circumpolar fronts are known to host (i) large densities of zooplankton and myctophids and (ii) that these organisms are patchily distributed^63^. In this study we suggest the potential mechanisms driving the patchiness observed at fine-scale. Assessing the preconditions and the other dynamics necessary for front aggregation is a new, open challenge emerging from the present work, whose findings look promising.

Note that in our work we focus only on open ocean Lagrangian fronts, that is, fine-scale frontal features induced by the mesoscale open ocean activity. In particular, by considering only the *Antarctic Southern Ocean* region defined by^41^, we intentionally exclude coastal fronts as well as large scale fronts. These can represent important productivity spots, and their dynamics and ecological role may be different (e.g.,^24,43,44^). Finally, the limitations of our analysis must be discussed. No vertical dynamics has been included in the model presented. However, these play an important role in the organization of marine biota^8^, and, typically, stronger gradients are present along the vertical scale^64^. This assumption is due to the fact that a 3D Lagrangian analysis, while appealing^65^, would add a level of complexity and a number of further parameters difficult to deal with, due to a lack of observational information on the vertical velocities of the ocean. Future satellite missions (such as SWOT: https://swot.cnes.fr/en) will possibly help to mitigate this problem, by helping the assimilation scheme to better reconstruct the three dimensional dynamics^66^. At the same time, they will also improve satellite resolution, providing a more precise location of fine-scale fronts.

The model studied does not consider the diel vertical migration of myctophids either. This choice is driven by the difficulty in parameterizing such a non linear behavior and in assimilating different migratory diel patterns. However, we note that many zooplankton species exhibit a diel cycle as well. Zooplankton consumption due to fish foraging is considered to be negligible, or compensated by source terms (such as rapid growth subsequent to a phytoplanktonic bloom). Finally, the density of fish predators in space and time, as well as their feeding frequency should be considered in future studies. The limitations presented can open the way for future investigations.

The results presented here may be useful for improving the representation of intermediate trophic levels in coupled ecological and physical models^60^, habitat models^67^, targeting the mesopelagic compartment in particular. At the same time, the possibility of using Lagrangian Coherent Structures combined with ocean colour information as a proxy of higher fish concentrations may further improve the integration of satellite-derived Lagrangian tools in conservation planning^68^.

## Supplementary Information

Supplementary Information.

## Data Availability

All data used in the present study are available upon request.
