# Supplementary material for: Fine-scale structures as spots of increased fish concentration in the open ocean
Source: Sci Rep. 2021 Aug 4;11:15805. doi: 10.1038/s41598-021-94368-1 (PMC8338936; doi:10.1038/s41598-021-94368-1)
Supplement: Supplementary file 1 — Supplementary Information. [file 41598_2021_94368_MOESM1_ESM.pdf]

Fine-scale structures as spots of increased fish concentration in the open ocean

Alberto Baudena[1,2,*], Enrico Ser-Giacomi[1], Donatella d'Onofrio[3,4], Xavier Capet[1], Cedric Cotté[1], Yves Cherel[5], and Francesco d'Ovidio[1]

[1] Sorbonne Université, CNRS, IRD, MNHN, Laboratoire d'Océanographie et du Climat: Expérimentations et Approches Numériques (LOCEAN-IPSL), Paris, France
[2]Sorbonne Université, Institut de la Mer de Villefranche sur mer, Laboratoire d'Océanographie de Villefranche, F-06230 Villefranche-sur-Mer, France
[3]Institute of Atmospheric Sciences and Climate, National Research Council (CNR-ISAC),Torino, Italy
[4]Copernicus Institute of Sustainable Development, Environmental Science Group, Utrecht University, The Netherlands
[5]Centre d'Etudes Biologiques de Chizé (CEBC), UMR 7372 du CNRS-La Rochelle Université, 79360 Villiers-en-Bois, France

[*] Corresponding author (email: alberto.baudena@gmail.com)

SUPPLEMENTARY INFORMATION

SI.1: Acoustic and satellite diagnostics: further analysis

Betweenness and Kinetic Energy

In this section we examine at the correlation between AFC and two further satellite derived diagnostics: betweenness and kinetic energy.
Betweenness is a local diagnostic inspired by Lagrangian flow network theory (1) that measures the amount of most probable paths crossing a given location of the seascape during a given time interval (2, 3, 4). High values of betweenness are associated with oceanic regions crossed by water masses coming from several origins and directed toward many different destinations. The average betweenness value (20.05) was used as the threshold for the bootstrap analysis. The Kinetic Energy (KE) was computed from velocity currents by KE=$\frac{1}{2}\left(u^2+v^2\right)$. The KE resolution is 0.25° and it was averaged over the 90 days preceding the acoustic measurements. The mean KE value (0.0092 m^2/s^2) was chosen as the threshold for the bootstrap analysis.

Fig. S.1 reports the scatter plots of the AFC against the betweenness and the KE. Red lines indicate linear quantile regression interpolations at the 75th, 90th, 95th and 99th percentiles. All quantile slopes are statistically significant. Fig. S.2 reports bootstrap results for the same diagnostics. These exhibit significantly stronger AFCs along features characterized by high values of betweenness. The KE instead exhibits the opposite trend.
Higher values of AFC indicate a positive trend for increasing values of FSLE, SST

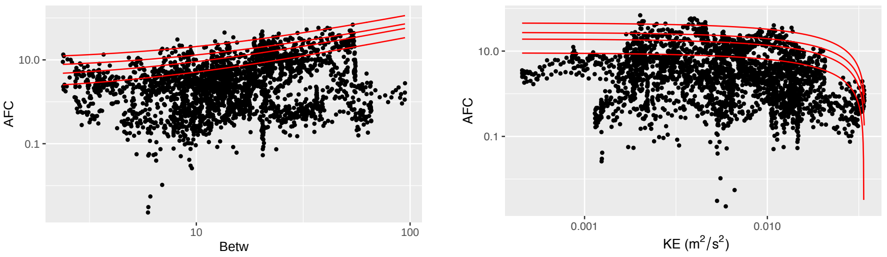

Fig. S.1: Scatterplots of AFC against two diagnostics. Left panel: betweenness computed over a period of 30 days. Right panel: Kinetic Energy, averaged over 90 days. Both axes are in the logarithmic scale. Each panel reports the linear quantile regressions at the 75th, 90th, 95th and 99th percentiles (red lines, respectively from the bottom to the top).

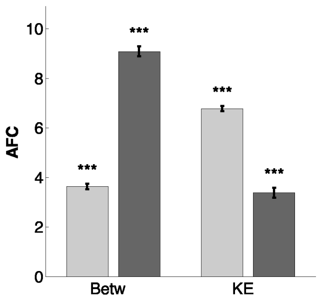

Fig. S.2: Bootstrap method results. Left columns: betweenness, computed over 30 days. Right columns: KE, averaged over 90 days. Methodologies are analog to those used in Fig. 2.

gradient and betweenness. For the sake of completeness, it must be observed that significantly high values of betweenness and SST gradient are associated only with small values of AFC (Fig. 3 and S.1). However, the portion of data following this trend is extremely reduced, being always smaller than 10% of the sample. Furthermore, the corresponding AFC are always extremely small. Considering that they are minor relative to the mean daily AFC, they are suspected to be simple noise. Finally, they were collected only over 2 days over the entire campaigns.

Linear quantile regression significance test

Fig. S.3 depicts the coefficients (intercept and slope) obtained with the quantile regression, when quantiles vary.
We tested the statistical significance of the quantile slopes (at the 99% confidence level) against the null-hypothesis of zero slope. We use a Monte-Carlo method,

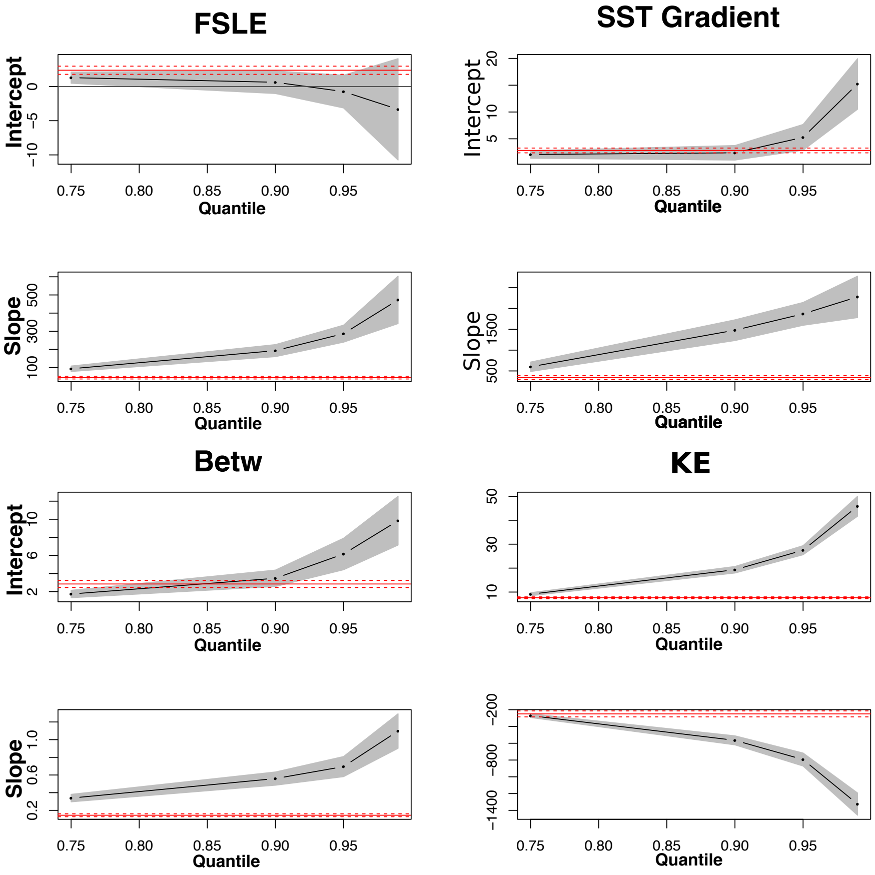

Fig. S.3: Linear quantile regressions analysis. Each couple of plots depicts the intercept and slope obtained when quantile varies. Gray bands indicate the confidence interval. Red lines indicate the results of the linear regression. Analyses were obtained with the QUANTREG package from R (5).

performed by randomly shuffling the satellite diagnostic data for a large number of times (1000 times in our case) (6, 7). The quantile slopes obtained from these randomized data were compared with the quantile slopes to be tested in a two-tailed test. Results are reported in Table S.1. All the quantile slope estimates were statistically significant for all the cases. Note that consistent results were also obtained using the t-test of the QUANTREG package (5).

Chlorophyll threshold sensitivity test

To select the chlorophyll-rich waters, we considered only the point which presented a local chlorophyll concentration above a certain threshold. In this subsection, we tested how the correlation coefficient (and the R^2) of the linear

		**Percentile**	**Slope value**	**Monte-Carlo test**	**t-test**
**FSLE**	Fig. 3	75	93.18	$< 10^{-5}$	$< 10^{-5}$
		90	193.21	$< 10^{-5}$	$< 10^{-5}$
		95	286.66	$< 10^{-5}$	$< 10^{-5}$
		99	472.88	$< 10^{-5}$	$< 10^{-5}$
**SST grad.**	Fig. 3	75	599.95	$< 10^{-5}$	$< 10^{-5}$
		90	1478.30	$< 10^{-5}$	$< 10^{-5}$
		95	1870.73	$< 10^{-5}$	$< 10^{-5}$
		99	2279.40	$< 10^{-5}$	$< 10^{-5}$
**Betw**	Fig. S.1	75	0.34	$< 10^{-5}$	$< 10^{-5}$
		90	0.56	$< 10^{-5}$	$< 10^{-5}$
		95	0.70	$< 10^{-5}$	$< 10^{-5}$
		99	1.10	$< 10^{-5}$	$< 10^{-5}$
**KE**	Fig. S.1	75	-270.37	$< 10^{-5}$	$< 10^{-5}$
		90	-564.94	$< 10^{-5}$	$< 10^{-5}$
		95	-793.82	$< 10^{-5}$	$< 10^{-5}$
		99	-1325.48	$< 10^{-5}$	$< 10^{-5}$

Table S.1: Linear quantile regression significance tests. The table reports the slope values (fourth column) obtained with the linear quantile regression, changing the diagnostics (indicated in the first column) and the quantiles (third column). The last 2 columns report, respectively, the significance tests obtained through the Monte-Carlo method (fifth column) and the t-test (sixth column). All the slopes obtained are significantly different from zero. Slope measure units are the following: days (FSLE), km/° (SST gradient), adimensional (betweenness), and s/m^2 (kinetic energy).

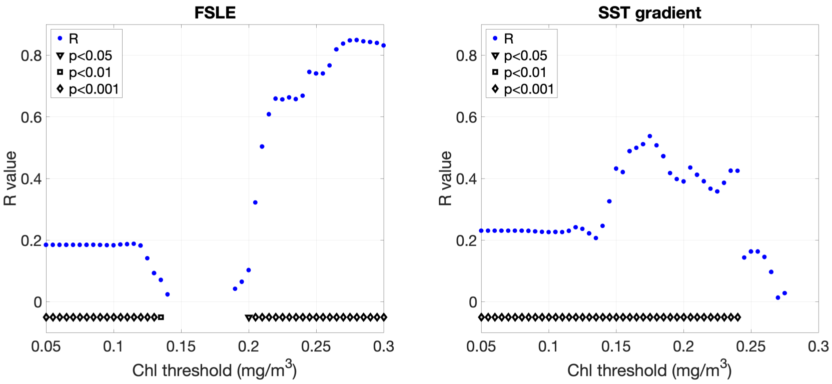

Fig. S.4: Coefficient of correlation (R, blue dots) obtained between AFC and FSLE (left panel) or SST gradient (right panel), when changing the chlorophyll threshold (x axis). The presence of a black symbol on the lower part of the panel indicates that the corresponding linear interpolation was significant (p<0.05, downward triangle; p<0.01, square; p<0.001, diamond).

interpolation between the AFC and the FSLE (or the SST gradient) changed when changing the chlorophyll threshold. The best correlation coefficients were obtained for values ranging between ∼0.20 and 0.25 mg/m^3 for the FSLE, and ∼0.160 and 0.220 mg/m^3 for the SST gradient (Fig. S.4). Values higher than 0.250 mg/m^3 were not considered since they excluded too many points (>95% of the dataset). Both the threshold intervals (for the FSLE and the SST gradient case) are similar. In addition, they are consistent with previous estimates used to characterise regions of enhanced primary production in the Southern Ocean (which were identified in 0.26 mg/m^3, 8). For these reasons, we considered a chlorophyll threshold of 0.22 mg/m^3 for the FSLE and of 0.17 mg/m^3 for the SST gradient.

Daytime data analysis

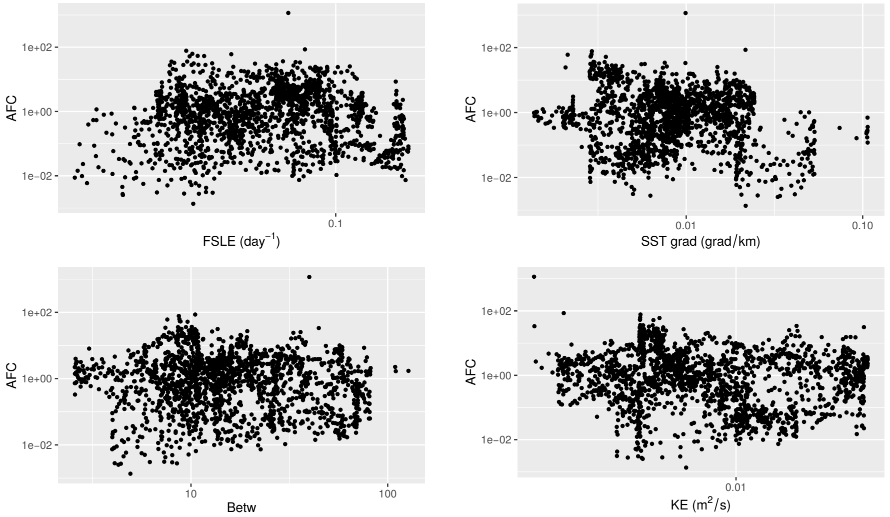

Fig. S.5: Scatterplots of AFC, measured during the day, against FSLE (top left panel), SST gradient (top right panel), betweenness (bottom left panel) and KE (bottom right panel). Both axes are in the logarithmic scale.

In this subsection, we display the analysis of the AFC measured during the day. Fig. S.5 depicts the scatter plots of the 4 diagnostics analyzed in the paper: FSLE, SST gradient, betweenness, and KE. The linear correlation coefficients between the AFC and the diagnostics are, for all the cases, smaller than 10^{-3}. All the diagnostics do not exhibit a significant bootstrap test (Fig. S.6). Therefore, quantile regression was not applied on daytime data.

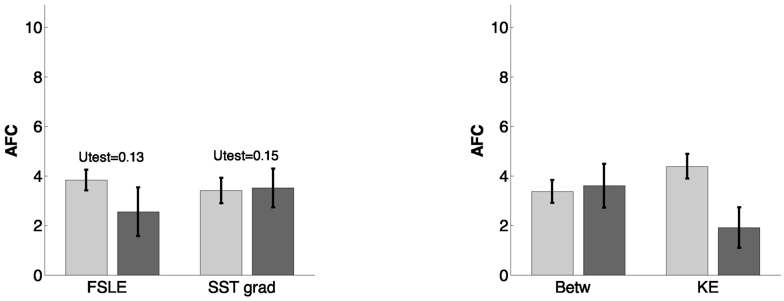

Fig. S.6: Bootstrap method results, computed with data measured during the day. Methodologies are analogous to those displayed in Fig. 2 and Fig. S.2. Left panel: FSLE and SST gradient. Right panel: betweenness, computed over 30 days and KE, averaged over 90 days.

SI.2 Orientating capacity of fish

In the present section, we aim to demonstrate the capacity of myctophids to identify, orientate, and follow a tracer cue in an aquatic environment, a central hypothesis in the present work. To do so, we will illustrate the latest developments on the knowledge about fish perceiving capacities. We focus in particular on their olfactive and visual sensibilities. We then review some advantages of living in schools in comparison to solitary individuals. Despite the lack of direct experimental data on myctophids, it is reasonable to conclude that, thanks to the available research on many other species, this family of fish is capable of identifying sources of food and following them even for several kilometers.

Current knowledge on fish orientating and feeding behavior. One of the most significant problems encountered by aquatic animals during foraging and migratory activities is the fact that the resources they need varies on large spatial scales (tens of kilometers), while their sensitive capabilities allow them to receive information on only a small volume of water around them (9, 10). This is valid in particular in the presence of noisy fluctuations overlying the gradient. However, even in apparently disadvantageous conditions, fish have developed fine-tuned sense capacities and social interactions that enable them to face these difficulties.

In fact, when examining fish behavior during the research in terms of feeding spots, two primary factors must be considered. The first one is the olfactory reception (11, 12, 13, 14), that, in fish, is involved in almost all behavioral forms, including spawning, locating food, homing, migrating, and defending. (15, 16, 17, 18, 19, 20). It is recognized that this system is even more essential for those species that base their feeding activities at night, thus in a dim environment, both for close and remote food searching activities (11).

The olfactory organ in fish is a paired structure located in most species on the

dorsal surface of the head. It is composed of a chemosensory epithelium inside an olfactory bulb. Its surface consists of folds, lamellae or rosettes, that host the receptor cells. The water that enters the olfactory bulb comes in contact with this rugose surface, and thus brings the substances dissolved in it into contact with the receptor cells (13, 11).
Fish possess an extremely high sensitivity to scents. The concentration threshold for perception (provoking significant electrophysiological responses) can have extremely low values (10^{-9}–10^{-13} M, where M is the molar concentration, *mol/l*). (21) demonstrated that a sensitivity of 10^{-11}M signifies that a salmonid fish is able to react to a single drop poured in a $25 \times 10 \times 2$m basin (500 000 liters). In some cases, even a few molecules can induce an electric response in the eel *Anguilla anguilla* (22).
These extremely high sensibilities allowed the fish to develop different mechanisms of orientation. The most important and widely accepted of these is the *klinotaxis*, by which a fish moves to the source of interest by swimming against the currents. It consists of a series of lateral movements, enabling the comparison of intensities of scent at different locations, which allows the fish to proceed along a gradient from low concentrations to higher ones (11). Among the other mechanisms of orientation are the *tropotaxis*, which is the comparison of scents perceived by the paired olfactory organs. However, for an efficient research with this method, the two organs need to be properly separated or a strong gradient must be present. Another mechanism is the *rheotaxis*, by which the fish turns to face to an oncoming current and keeps its position rather that being advected by the current (13).

A second element to consider when analyzing the food seeking strategies of fish is the fact that they typically live in schools. Schools are groups of aquatic animals of the same species that move together, displaying strong synchronicity in behavior and a large range of adaptive functions (23, 24, 25, 26). Different dynamics can lead a fish school to climb gradients more efficiently than individuals. One of them is the so-called "imitation behavior", a key ability of fish school formation, by which a fish tends to copy the behavior of a neighbor. This can lead a school of fish to reach a feeding spot that they would have not been capable of reaching alone (27). Another interesting dynamic occurs during exploratory behavior, in which some fish leave the school and rapidly return several times. When a source of food is identified, this movements repeats with more fish and for larger distances. This leads to the formation of "tentacle" structures that can disappear or improve in size. In fact, under favorable conditions, the fish can eventually propel themselves in these tentacles that will thus inflate and form the new school (26). This mechanism demonstrates how collective research can help averaging the environmental noise and improve the gradient climbing capacity of schools compared to isolated individuals. Other mechanisms leading in this direction have been identified theoretically and experimentally (28, 29, 25, 30, 31).
Finally, some studies have highlighted the hydrodynamical advantages of swimming in schools. Even if the mechanisms of the hydrodynamic interrelationship

between the fish are poorly understood, some indirect proofs indicate that there is an energy saving mechanism when displacing inside a school rather than outside. Experiments reported that some schools in facts are capable of maintaining cruising speeds 3-5 times higher than individuals, with higher endurance (32). Furthermore, it has been demonstrated that the lead fish of a school consumes more energy than the others because of higher caudal movements (33).

Focus on myctophids The studies discussed in the previous subsection illustrate the strong capacities of fish in orientating and climbing gradients even at large scales. Despite the relative lack of studies on myctophids concerning these issues (see for instance (34) about school behavior in lanternfishes), (35) has described the olfaction organ in *Tarletonbeania crenularis*, a lanternfish. The description of the organ and its epithelium does not differ substantially from the classical one given in the previous section. Furthermore, myctophids are a worldwide diffused fish and their biomass is one of the greatest in the Southern Ocean, constituting the dominant portion of the biomass (36, 37). Myctophids are constituted by more than 250 species in 33 genera and two subfamilies (38), and they have diversified at higher rates than other families (39). Some authors have theorized that this diversification has been possible due to greater adaptive capacities. In particular, (40) has demonstrated the great visual capacity of lanternfishes by measuring their optical sensitivity with the Land formule for monochromatic light (41, 42). The authors observed that myctophids eyes are approximately 10-100 times more sensitive than human eyes.
It is thus reasonable to conclude that myctophids capacities do not differ significantly from those of other fish discused in the previous subsection. On the contrary, the dim-light environment in which they live and forage, along with their success in spreading worldwide and diversifying, suggest that they may possess significant capacities to adapt to various unique and hostile environments, both physiologically and behaviorally. This is why, in the present work, we assumed that myctophids are capable of detecting a tracer and trying to climb or follow it.

SI.3: Gradient Climbing Model, behavior 2

In this section, the gradient climbing model is described. The results of this model are tested via a different fish behavior.
We assume that, at each timestep, the fish is able to observe the tracer only along a random direction (in our case, right or left). The tracer T is affected by a noise term. The effective value perceived by the fish will be:

$$\tilde{T}(x_0 \pm \Delta x) = T(x_0) \pm p_R \frac{\partial T}{\partial x} + \xi \ .$$

We assumed that, if $\tilde{T}(x_0 \pm \Delta x) > T(x_0)$, the fish will move in the observed position $x_0 \pm \Delta x$, and, otherwise, will stay in the original position x_0.
In case of absence of a noise term, or with $\xi_{MAX} < p_R \frac{\partial T}{\partial x}$, the fish will always

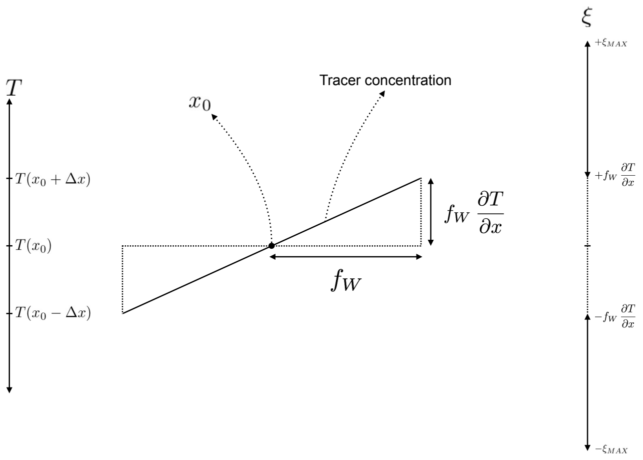

Fig. S.7: Schematic representation of the behavior of a fish. Each timestep, the fish can observe the tracer, according to its perceptual range capacity, up to a distance p_R. We assume a small field view, so that the tracer variation can be considered linear, and a positive gradient. The tracer value in the new position is affected by a noise term, ranging between $-\xi_{MAX}$ and ξ_{MAX}. Behavior 1: the fish observes both directions, and swims toward the one with a higher tracer. Behavior 2: if the fish observes a tracer value higher in a randomly selected direction, it will move there; otherwise, it will stay in the actual position x_0.

stay right in case of rightward observation, and will not move in case of leftward observation. Considering that the probability of observing right or left (P_{OR} and P_{OL} respectively) is equal to $\frac{1}{2}$, the average velocity of the fish school $U_F(x)$ will be $\frac{1}{2}V$.

Now, assume $\xi_{MAX} > p_R \frac{\partial T}{\partial x}$. We will have the following scenario:

Observation on the right:
if $\xi > 0 \rightarrow$ the fish moves right
if $-p_R \frac{\partial T}{\partial x} < \xi < 0 \rightarrow$ the fish moves right
if $-\xi_{MAX} < \xi < -p_R \frac{\partial T}{\partial x} \rightarrow$ the fish does not move.

Observation on the left:
if $\xi < 0 \rightarrow$ the fish does not move
if $0 < \xi < +p_R \frac{\partial T}{\partial x} \rightarrow$ the fish does not move
if $+p_R \frac{\partial T}{\partial x} < \xi < +\xi_{MAX} \rightarrow$ the fish moves left.

The probability of moving right is given by the product of P_{OR} and the probability that the noise will lead to a rightward movement. The latter is the ratio between the length of the interval of noise for which the fish remains in the new

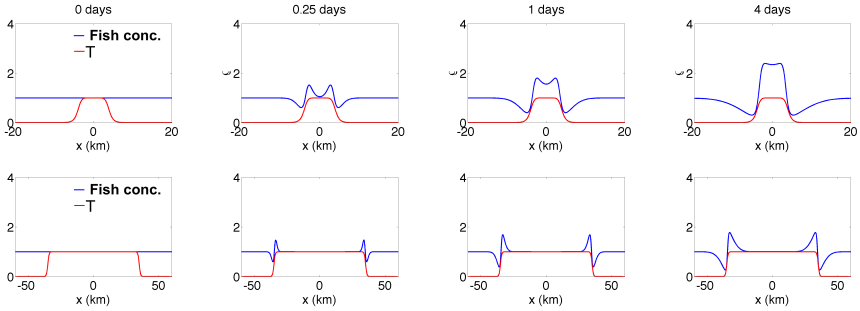

Fig. S.8: Time evolution of the fish concentration (blue line, adimensional) according to the continuity equation. Upper row: the tracer (red line, adimensional) describes a plateau of 8 km width. At its limits, its values range from 1 to 0 in about 5 km. Each panel represents a different snapshot: at 0, 6 hours, 1 day, and 4 days. Second row: same evolution as in the upper row. This time the plateau width was set set to 70 km.

position and the range of the noise. The procedure is analogous for $P(L)$. Thus:

$$P(R) = P_{OR} * \frac{1}{2\xi_{MAX}}[\xi_{MAX} + p_R \frac{\partial T}{\partial x}]$$
$$P(L) = P_{OL} * \frac{1}{2\xi_{MAX}}[\xi_{MAX} - p_R \frac{\partial T}{\partial x}]$$

,

and their difference:

$$P(R) - P(L) = \frac{p_R}{2\,\xi_{MAX}} \frac{\partial T}{\partial x} \tag{S.1}$$

gives the frequency of rightward movement. Thus, the average velocity of the fish school $U_F(x)$ will be:

$$U_F(x) = \frac{1}{2} \frac{V p_R}{\xi_{MAX}} \frac{\partial T}{\partial x} \,. \tag{S.2}$$

Substituting then Expr. (2) into Expr. (S.2):

$$U_F(x) = \frac{1}{2} V \frac{\frac{\partial T}{\partial x}}{\frac{\partial T}{\partial x}_{MAX}} \,. \tag{S.3}$$

For this case, we can also take into account the effect of the currents by adding their speed U_C to $U_F(x)$.

Results. The continuity equation (described in Materials and Methods) is simulated using the Lax method (43). The choice of the physical and biological parameters is described in SI.6. Results are reported in Fig. S.8. The main difference between results depicted in Fig. 5 and 6 is represented by the intensity

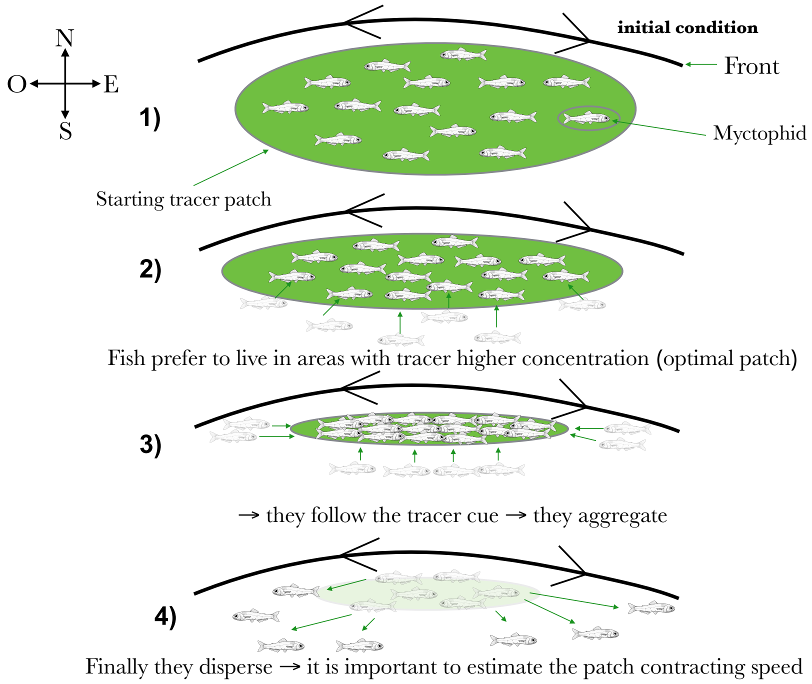

Fig. S.9: Illustrative scheme of the hyperbolic dynamic analyzed: the green patch represents an optimal region for fish to live in, while the black line indicates the front, which correspond to a stretching region. After a certain amount of time, the patch will be compressed along the N-S direction and enlarged along the W-E due to stirring. Then, due to the diffusion effect, the patch will also begin to reduce in this direction. If the fish have the swimming capability to follow the patch, their local density will improve and thus they will aggregate. When the green patch disappears, the fish disperse again.

of the concentrations. For the second type of behavior, peak concentrations are lower. This is not surprising, since behavior 2 implies a weaker fish orientation capacity (observation along a random direction, and not on both the possible ones). However, the trend is strongly similar to that depicted in Fig. 5 and 6.

SI.4: Hyperbolic dynamic aggregating mechanism

The results reported in Fig. 5, 6 and in section SI.3 are identical if the whole system (fish+tracer) is transported leftward or rightward by a uniform current $U_C(x)$ =const. However, this is typically not the case for submesoscale frontal features, which are characterized by high stirring rates and in which diffusion plays a consistent role (44). A typical dynamic of this type is the one represented by the so-called hyperbolic points (45, 46, 47). These are characterized by a compression-elongation dynamic along two stretching directions, called respectively the stable and unstable manifold. A patch of tracer in the proximity

of this structure is typically compressed along the stable manifold and elongated along the other. Eventually, diffusion acts on the thinning of the tracer by dissipating it and reducing its concentration until it is below the threshold detected by the fish. Note that due to the effect of diffusion an isoconcentration contours of the tracer eventually retracts, even if the tracer is stretched along the unstable manifold. If we suppose that fish are interested in the region with high values of the concentration, they will swim to follow the contracting patch, and thus their concentration will improve (Fig. S.9).

Do myctophids have swimming capabilities strong enough to counter the dynamics of the reducing patch? We address this question by estimating the speed of an isoconcentration contour of a passive tracer under typical diffusion and stretching conditions of the Southern Ocean.

We analyze the evolution of a patch in the reference system of its center of mass over an unstable manifold. In this case, the velocity field can be written as:

$$\begin{aligned} u &= +\lambda x \\ v &= -\lambda y \end{aligned} \tag{S.4}$$

in which λ is the strain rate given by the Lyapunov exponents.

Taking into consideration the horizontal diffusivity k, the patch, represented by a tracer concentration T, evolves in time following the advection-diffusion equation. The latter, considering the velocity field given by (S.4) can be written as:

$$\frac{\partial T}{\partial t} + \lambda x \frac{\partial T}{\partial x} - \lambda y \frac{\partial T}{\partial y} \;=\; k \left(\frac{\partial^2 T}{\partial x^2} + \frac{\partial^2 T}{\partial y^2} \right) \;+\; \mathcal{S}\,. \tag{S.5}$$

Neglecting eventual source or sink terms, represented by $\mathcal{S}$, and assuming T as gaussian, Eq. (S.5) can be solved analytically:

$$T(x, y, t) \;=\; \frac{M}{\sigma_u(t)\,\sigma_s(t)}\, exp\left[-\frac{x^2}{2\sigma_u^2(t)} - \frac{y^2}{2\sigma_s^2(t)} \right] \tag{S.6}$$

with M the total mass of the tracer and

$$\begin{aligned} \sigma_u^2(t) \;&=\; \left(\sigma_{u_0}^2 + \frac{k}{\lambda} \right) e^{2\lambda t} - \frac{k}{\lambda} \\ \sigma_s^2(t) \;&=\; \left(\sigma_{s_0}^2 - \frac{k}{\lambda} \right) e^{-2\lambda t} + \frac{k}{\lambda} \end{aligned} \tag{S.7}$$

that are, respectively, the standard deviation of the tracer concentration along the unstable and stable manifold. We assume $M = \sigma_{u_0} \sigma_{s_0}$, so that the maximum starting value of the distribution (adimensional) is 1.

We define as *optimal patch* for the fish the ensemble of the points with tracer concentration greater than a certain threshold T_t (their detection threshold), $0 < T_t < 1$. The position of the points with tracer concentration $T = T_t$ therefore delimit the *optimal patch.* Their positions, along the stable ($x = 0$) and unstable manifold ($y = 0$), can be obtained by inverting Eq. (S.6):

$$p_u(t) = \sqrt{2\sigma_u^2(t) Log\left(\frac{M}{\sigma_u(t)\sigma_s(t)T_t}\right)} \tag{S.8}$$

$$p_s(t) = \sqrt{2\sigma_s^2(t) Log\left(\frac{M}{\sigma_u(t)\sigma_s(t)T_t}\right)}\,, \tag{S.9}$$

and their velocities $\dot{p}_u(t)$, $\dot{p}_s(t)$, deriving Eq. (S.8) and (S.9) in time (not reported). $\dot{p}_u(t)$ and $\dot{p}_s(t)$ thus represent the expanding (positive values) or contracting (negative values) speed of the boundaries of the *optimal patch.* Since the myctophids swim in a current flow, we define their absolute swimming speed S_{as} as:

$$S_{as} = V + U_C$$

in which $U_C = -\lambda x$ along the unstable manifold, and $+\lambda y$ along the stable one, and V is the myctophids cruising swimming speed. We evaluate the S_{as} at the limits of the *optimal patch*, thus

$$x = p_u(t) \qquad\qquad y = p_s(t)\,. \tag{S.10}$$

The choice of physical and biological parameters is described in SI.6, and the results are depicted in Fig. S.10. Along the unstable manifold (panel A), the *optimal patch* expands for the first two weeks, then it decreases until it disappears after a “lifetime” of 17.65 days. The fish have a S_{as} greater than $\dot{p}_u(t)$ (in absolute value) for $t_u = 17.49$ days (panel B). Along the stable manifold (panel C), the *optimal patch* decreases regularly for all the periods, and the S_{as} is greater than $\dot{p}_s(t)$ (in absolute value) for $t_s = 17.64$ days (panel D). We define the “fish concentration life time” (FCLT) as the minimum between t_u and t_s. This is because it indicates the first moment after which a part of the school can not follow any more the *optimal patch.* After this amount of time, the fish school can potentially disintegrate. The situation of the tracer at t=FCLT is reported in Fig. S.10, panel E, with the contour identifying the *optimal patch.* In panel F we display the aggregation estimate $\mathcal{A}$, defined as the ratio between the starting surface of the *optimal patch* and its value after a time t:

$$\mathcal{A}(t) = \frac{p_u(0)\,p_s(0)}{p_u(t)\,p_s(t)} \tag{S.11}$$

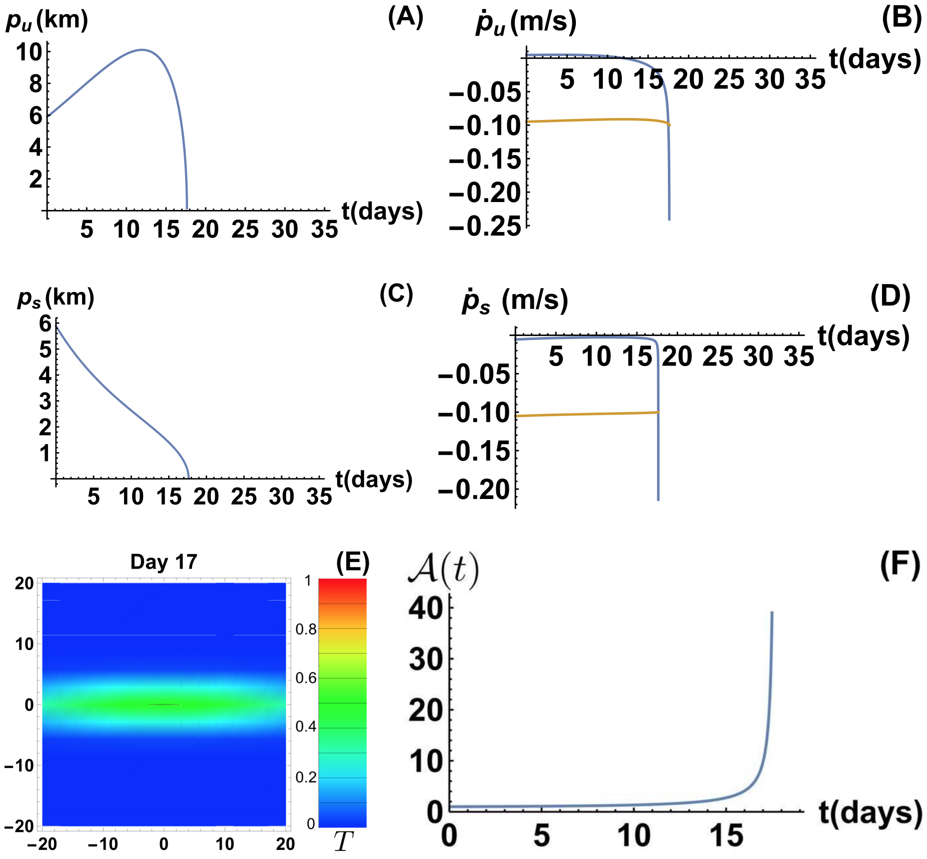

Fig. S.10: *Optimal patch* boundary position (p) and velocity ($\dot{p}$) along the unstable (A-B) and stable (C-D) manifold, according to the advection-diffusion equation (Eq. S.5). K=4 m^2/s, $\lambda = 0.1$ days^{-1}, T with starting gaussian concentration with $\sigma_{u_0} = \sigma_{s_0} = 5$ km. T_t=0.5. In the plots reporting $\dot{p}_u$ and $\dot{p}_s$ (panels B and D), the orange line represents the absolute swimming speed of the fish (S_{as}). Panel E: *optimal patch* (delimited by the gray thin line) evolution at t=FCLT: for about 17 days, the fish have a S_{as} greater than $\dot{p}$ along both the directions, thus potentially aggregating. This is demonstrated in panel F, which illustrates the evolution of the aggregating estimate $\mathcal{A}$ (Eq. S.11) in time. The starting fish aggregation value is 1.

with $0 < t <$FCLT. The aggregation $\mathcal{A}$ improves slowly in the first two weeks, reaching a value of ~ 4 times after 15 days, and then improves dramatically in the last 2 days of FCLT, reaching a maximum value of 39 times the starting aggregation.

We note that Expr. (S.10) is a limiting aggregating condition, because it assumes that fish swim only if they are at the boundaries of the *optimal patch*, and not always toward the area of maximum tracer concentration. Nevertheless, it is

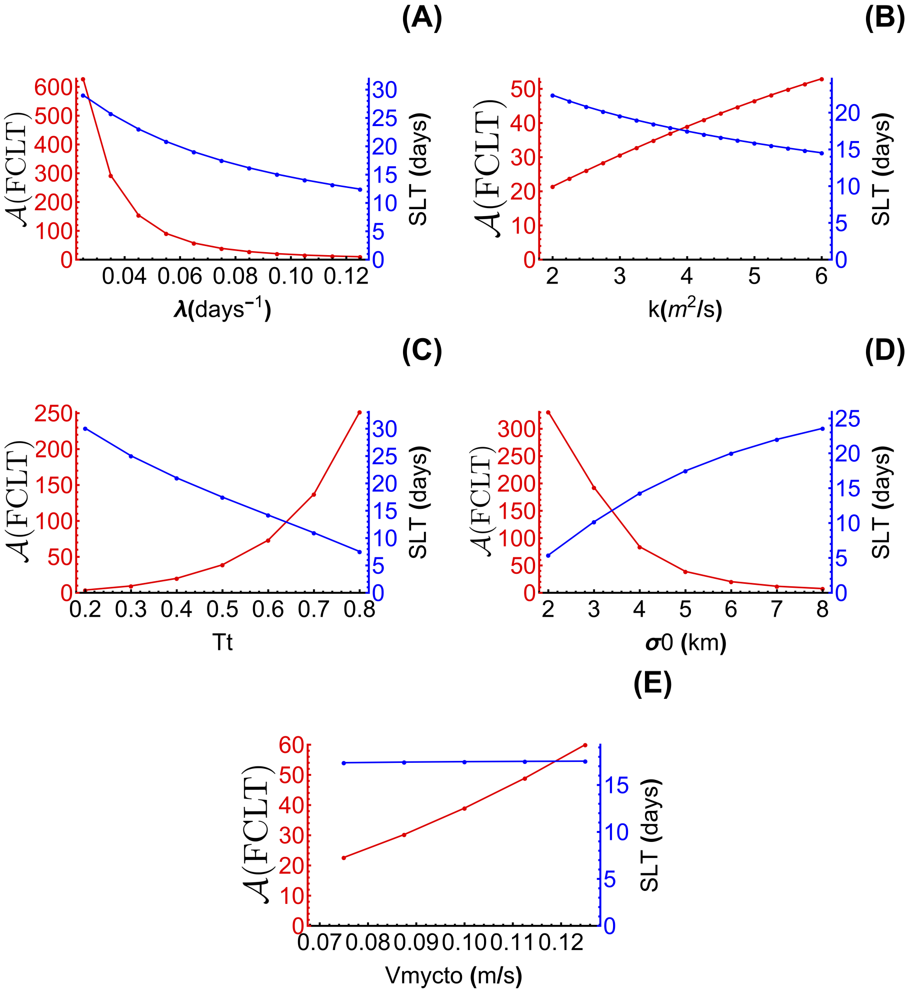

Fig. S.11: Sensitivity test results on the 5 variables used to study FCLT and $\mathcal{A}$(FCLT). These are: λ (panel A), k (panel B), T_t (panel C), σ_0 (panel C) and V (panel E). For each panel, two y axes are present: the left red axis, which refers to $\mathcal{A}$(FCLT) values (in red), and the right blue axis, which refers to FCLT values (in blue).

possible to assume that fish continuously swim, as described in the previous subsection: in that case, their aggregation could improve more (not displayed).
Sensitivity test. The extent to which the fish aggregation varies according to the different parameters used was also tested. Five variables were tested: the Lyapunov exponent λ, the diffusion coefficient k, the threshold of the *optimal patch* T_t, the initial tracer standard deviation σ_0, and the myctophids' cruising speed V.
Each variable was tested independently: each time, 4 variables were fixed, using

the values described in SI.6. We then varied the variable left over a series of values. These ranged between about 0.5 and 1.5 times the value described in SI.6. For each test value chosen, we computed the FCLT and $\mathcal{A}$(FCLT). These are reported in Fig. S.11 and discussed below.

- λ test (panel A). FCLT and $\mathcal{A}$(FCLT) decrease while λ increases. This implies that stronger fronts aggregate less. Thus, one may expect that weaker structures can aggregate more. However, when examining this sensitivity test result, some important considerations must be made. In this regard, stronger fronts aggregate less, but on shorter timescales (FCLT decreases as λ increases). In the ocean, weak fronts have shorter lifetimes. Thus, they can not preserve the hyperbolic dynamic for a consistent amount of time necessary for the aggregation.
- k test (panel B). As diffusion increases, FCLT diminishes. $\mathcal{A}$(FCLT) is instead enhanced because the size of the *optimal patch* is kept smaller. Thus fish do not encounter stronger currents, according to Expr. S.4, and therefore have more time to aggregate. For higher k values (around 12 m^2/s), $\mathcal{A}$(FCLT) begins to diminish because of the small FCLT (not depicted in the graphic).
- T_t test (panel C). Increasing the tracer threshold obviously diminishes the FCLT. Smaller *optimal patches* will have shorter life times. Interestingly, this enhances $\mathcal{A}$. However, we note that $\mathcal{A}$ is relative to the starting *optimal patch* size.
- σ_0 test (panel D). Increasing the starting size of the *optimal patch* increases its lifetime, and therefore FCLT. According to Expr. S.4, stronger currents are encountered by the fish. This implies a weaker S_{as}, which results in a decreased $\mathcal{A}$.
- V test (panel E). Not surprisingly, a stronger cruising myctophids speed results in a stronger aggregation $\mathcal{A}$(FCLT). FCLT is rather almost constant because of the quick contraction of the *optimal patch* at the end of its life time.

Since the *optimal patch* tends to disappear shortly after the FCLT, we consider it a proxy of the *optimal patch* life time. We obtain an average life time of about two weeks.

SI.5: Zooplankton gradient computation.

Zooplankton concentration and zooplankton gradient computation

The zooplankton concentration was inferred from a set of data collected within the Mycto-3D-MAP program between January the 22nd and February the 5th, 2014, during a dedicated cruise in the Kerguelen region. It was processed in the same manner as the first dataset described in Materials and Methods (subsection "Acoustic measurements"), but this time the bifrequency algorithm was used to infer the zooplankton biomass. This set possesses a high resolution with an acoustic unit every 206 m of the ship trajectory on average.

The dataset was used to infer the Acoustic Zooplankton Concentration (AZC) in the water column. We considered as AZC of the point (x_i, y_i) the average of the bifrequency acoustic backscattering on the whole column, with the exclusion of the first layer. The latter was not considered due to surface noise. AZC quantity is dimensionless.
The AZC was used to compute the zooplankton gradient. The zooplankton gradient of a point i of the ship transect is computed as:

$$\frac{\partial Z}{\partial x}(i) = \frac{1}{2}\left(\frac{Z_i - Z_{i-1}}{d_{i-1}} + \frac{Z_{i+1} - Z_i}{d_i}\right)$$

in which d_i indicates the kilometric distance between the point $i+1$ and i, and Z_i is the cubic spline interpolation (to smooth the noise effects), in an around of 2 km, of the zooplankton concentrations. This type of interpolation, in contrast with the moving average, preserves the trend of the data, and thus a possible front. AZC was not compared to the AFC. This is due to the fact that the main size of the zooplankton detected by the 120 kHz frequency of the acoustic sonar is too large compared to the size of the prey of myctophids, for which a 200 kHz echo sounder would have been necessary.

SI.6: Estimation of physical and biological parameters.

Gradient Climbing Model. As an expression of the tracer concentration T, we use a symmetric sigmoid function, defined as:

$$T(x) = \frac{1}{1 + \left(\frac{x-c}{a}\right)^{\frac{10a}{f}}} \tag{S.12}$$

in which $c = 0\,\mathrm{m}$ is the centre of the curve, $2a = 8$ km is the width of the plateau, and f the width of the front, or the region characterized by a gradient in the tracer. We consider a window with periodic boundary conditions (ranging between [-20 +20] or [-70 +70]km). The integrating time step is $\Delta t = 1$s. The spatial separation between two contiguous points is $100\,\Delta t\,V$ to respect the Courant condition.
$\frac{\partial T}{\partial x}_{MAX}$ is determined from the 90th percentile (the choice of 90th percentile is arbitrary) of the distribution of the normalized zooplankton gradient. This was computed as described in the Materials and Methods section. The obtained value is $\frac{\partial T}{\partial x}_{MAX} = 0.3475$ km^{-1}.
We employed a cruising swimming speed V of about one body length per second (48, 49, 50, 51). Because the reference fish are the myctophids, whose size is about 10 cm ((36) and Andrea Walters, pers. comm.),V was set to 0.1 $\frac{m}{s}$.
Hyperbolic dynamic aggregating mechanism. 5 variables were parameterized: λ, k, T_t, σ_0 and V. The latter was estimated as explained in the previous

subsection ($V = 0.1\,\frac{m}{s}$).
A typical value of Lyapunov exponent calculated using satellite-derived velocity currents is of the order of 0.1 days^{-1} (see for instance 52). This value is representative of the ocean upper layer. Since myctophids do not live in the mixed layer, we followed the indications in (53) for the estimation of the Lyapunov exponents in the column water. Therefore, we consider λ to be 75% of the surface value, or $\lambda = 0.075$ days^{-1}, which is indicative of a depth of $\sim$ 200 m.
As a value of horizontal diffusivity, we utlized the average value $k = 4\,\frac{m^2}{s}$. This is obtained from the estimates across an ocean front, with the Lyapunov intensity corresponding with (44).
The optimal patch threshold T_t was set to 0.5. The choice of the value is arbitrary but it is tested in SI.4.
Finally, we employed a patch of $\sigma_{u_0} = \sigma_{s_0} = 5$ km, typical fine-scale range.

Bibliography

[1] E. Ser-Giacomi, V. Rossi, C. López, E. Hernández-García, *Chaos: An Interdisciplinary Journal of Nonlinear Science* **25**, 036404 (2015).

[2] E. Ser-Giacomi, R. Vasile, E. Hernández-García, C. López, *Physical Review E* **92**, 012818 (2015).

[3] E. Ser-Giacomi, R. Vasile, I. Recuerda, E. Hernández-García, C. López, *Chaos: An Interdisciplinary Journal of Nonlinear Science* **25**, 087413 (2015).

[4] E. Ser-Giacomi, *et al.*, From network theory to dynamical systems and back: Lagrangian betweenness reveals bottlenecks in geophysical flows (2019).

[5] R Core Team, *R: A Language and Environment for Statistical Computing*, R Foundation for Statistical Computing, Vienna, Austria (2015).

[6] E. Pollard, K. H. Lakhani, P. Rothery, *Ecology* **68**, 2046 (1987).

[7] N. Ciccarelli, *et al.*, *Global and Planetary Change* **63**, 185 (2008). Mediterranean climate: trends, variability and change.

[8] S. Sergi, *et al.*, *Frontiers in Marine Science* **7**, 416 (2020).

[9] J. Atema, *Sensory biology of aquatic animals* **1**, 29 (1988).

[10] W. F. Royce, *Introduction to the fishery sciences* (Academic Press, 2013).

[11] A. Kasumyan, *Journal of Ichthyology* **44**, S180 (2004).

[12] R. J. Grimm, *Science* **131**, 162 (1960).

[13] T. J. Hara, *Progress in neurobiology* **5**, 271 (1975).

[14] A. O. Kasumyan, K. B. Döving, *Fish and fisheries* **4**, 289 (2003).

[15] H. Kleerekoper, *Indiana University Press* (1969).

[16] G. Malyukina, N. Dmitrieva, E. Marusov, G. Yurkevich, *Itogi Nauki, Ser. Biol. Zool* pp. 32–78 (1969).

[17] G. Malyukina, A. Kasumyan, E. Marusov, *Sensory Systems* pp. 30–44 (1980).

[18] K. Døving, *Progress in Sensory Physiology 6* (Springer, 1986), pp. 39–104.

[19] E. H. Hamdani, A. Kasumyan, K. B. Døving, *Chemical senses* **26**, 1133 (2001).

[20] A. Kasumyan, E. Marusov, *Journal of Ichthyology* **43**, 528 (2003).

[21] K. B. Døving, *Molecules in physics, chemistry, and biology* (Springer, 1989), pp. 299–329.

[22] H. Teichmann, *Zeitschrift für vergleichende Physiologie* **42**, 206 (1959).

[23] D. V. Radakov (1973).

[24] T. J. Pitcher, *The behaviour of teleost fishes* (Springer, 1986), pp. 294–337.

[25] D. Grünbaum, *Evolutionary Ecology* **12**, 503 (1998).

[26] D. Pavlov, A. Kasumyan, *Journal of Ichthyology* **40**, S163 (2000).

[27] D. Radakov, *Nauka, Moscow* (1972).

[28] E. Shaw, B. D. Sachs, *Journal of comparative and physiological psychology* **63**, 385 (1967).

[29] A. Okubo, *Advances in biophysics* **22**, 1 (1986).

[30] P. Ogren, E. Fiorelli, N. E. Leonard, *IEEE Transactions on Automatic control* **49**, 1292 (2004).

[31] T. Vicsek, A. Zafeiris, *Physics Reports* **517**, 71 (2012).

[32] C. Breder, *Zoologica* **52**, 25 (1967).

[33] J. Herskin, J. Steffensen, *Journal of Fish Biology* **53**, 366 (1998).

[34] R. A. Saunders, S. Fielding, S. E. Thorpe, G. A. Tarling, *Deep Sea Research Part I: Oceanographic Research Papers* **81**, 62 (2013).

[35] J. V. Lawry, *Cell and Tissue Research* **138**, 31 (1973).

[36] E. Pakhomov, R. Perissinotto, C. McQuaid, *Marine Ecology Progress Series* pp. 1–14 (1996).

[37] Y. Cherel, C. Fontaine, P. Richard, J.-P. Labatc, *Limnology and oceanography* **55**, 324 (2010).

[38] V. Catul, M. Gauns, P. K. Karuppasamy, *Reviews in Fish Biology and Fisheries* **21**, 339 (2011).

[39] M. P. Davis, N. I. Holcroft, E. O. Wiley, J. S. Sparks, W. L. Smith, *Marine biology* **161**, 1139 (2014).

[40] F. de Busserolles, N. J. Marshall, *Phil. Trans. R. Soc. B* **372**, 20160070 (2017).

[41] T. W. Cronin, S. Johnsen, N. J. Marshall, E. J. Warrant, *Visual ecology* (Princeton University Press, 2014).

[42] F. De Busserolles, J. L. Fitzpatrick, N. J. Marshall, S. P. Collin, *PloS one* **9**, e99957 (2014).

[43] W. H. Press, S. A. Teukolsky, W. T. Vetterling, B. P. Flannery, *Cambridge University Press* **1**, 3 (1988).

[44] F. Nencioli, F. d'Ovidio, A. M. Doglioli, A. A. Petrenko, *Journal of Geophysical Research: Oceans* **118**, 7066 (2013).

[45] G. Haller, *Physics of Fluids* **13**, 3365 (2001).

[46] A. Mancho, D. Small, S. Wiggins, *Nonlinear Processes in Geophysics* **11**, 17 (2004).

[47] Y. Lehahn, F. d'Ovidio, M. Lévy, E. Heifetz, *Journal of Geophysical Research* **112**, C08005 (2007).

[48] R. Bainbridge, *Journal of experimental biology* **35**, 109 (1958).

[49] M. Sfakiotakis, D. M. Lane, J. B. C. Davies, *Oceanic Engineering, IEEE Journal of* **24**, 237 (1999).

[50] P. Domenici, *Comparative Biochemistry and Physiology Part A: Molecular & Integrative Physiology* **131**, 169 (2001).

[51] F. Gui, P. Wang, C. Wu, *Agricultural Sciences* **5**, 106 (2014).

[52] F. d'Ovidio, *et al.*, *Biogeosciences* **12**, 5567 (2015).

[53] J. H. Bettencourt, C. López, E. Hernández-García, *Ocean Modelling* **51**, 73 (2012).
